# Evidence Update on the Relationship between Diet and the Most Common Cancers from the European Prospective Investigation into Cancer and Nutrition (EPIC) Study: A Systematic Review

**DOI:** 10.3390/nu13103582

**Published:** 2021-10-13

**Authors:** Esther Ubago-Guisado, Miguel Rodríguez-Barranco, Ana Ching-López, Dafina Petrova, Esther Molina-Montes, Pilar Amiano, Aurelio Barricarte-Gurrea, María-Dolores Chirlaque, Antonio Agudo, María-José Sánchez

**Affiliations:** 1 Cancer Registry of Granada, Escuela Andaluza de Salud Pública, 18011 Granada, Spain; esther.ubago@gmail.com (E.U.-G.); ana.ching.easp@juntadeandalucia.es (A.C.-L.); dafina.petrova.easp@juntadeandalucia.es (D.P.); mariajose.sanchez.easp@juntadeandalucia.es (M.-J.S.); 2 Epidemiology and Control of Chronic Diseases, CIBER of Epidemiology and Public Health (CIBERESP), 28029 Madrid, Spain; memolina@ugr.es (E.M.-M.); epicss-san@euskadi.eus (P.A.); aurelio.barricarte.gurrea@navarra.es (A.B.-G.); mdolores.chirlaque@carm.es (M.-D.C.); 3 Cancer Epidemiology Group, Instituto de Investigación Biosanitaria ibs.GRANADA, 18012 Granada, Spain; 4 Department of Experimental Psychology, Mind, Brain and Behavior Research Center (CIMCYC), University of Granada, 18071 Granada, Spain; 5 Department of Nutrition and Food Science, Campus of Cartuja, University of Granada, 18071 Granada, Spain; 6 Institute of Nutrition and Food Technology (INYTA) ‘José Mataix’, Biomedical Research Centre, University of Granada, Avenida del Conocimiento s/n, E-18071 Granada, Spain; 7 Public Health Division of Gipuzkoa, BioDonostia Research Institute, 20014 Donostia-San Sebastian, Spain; 8 Navarra Public Health Institute, 31008 Pamplona, Spain; 9 Navarra Institute for Health Research (IdiSNA), 31008 Pamplona, Spain; 10 Department of Epidemiology, Regional Health Council, IMIB-Arrixaca, Murcia University, 30003 Murcia, Spain; 11 Unit of Nutrition and Cancer, Catalan Institute of Oncology—ICO, 08908 L’Hospitalet de Llobregat, Spain; a.agudo@iconcologia.net; 12 Nutrition and Cancer Group, Epidemiology, Public Health, Cancer Prevention and Palliative Care Program, Bellvitge Biomedical Research Institute—IDIBELL, 08908 L’Hospitalet de Llobregat, Spain; 13 Department of Preventive Medicine and Public Health, University of Granada, 18071 Granada, Spain

**Keywords:** colorectal cancer, breast cancer, lung cancer, prostate cancer, intake, alcohol, fruits, vegetables, meat, fish

## Abstract

The European Prospective Investigation into Cancer and Nutrition (EPIC) is a multicentre prospective study conducted in 23 centres in 10 European countries. Here we review the findings from EPIC on the relationship between diet-related exposures and incidence or mortality from the four most frequent cancers in the European population: colorectal, breast, lung, and prostate cancer. We conducted a systematic review following PRISMA guidelines and identified 110 high-quality studies based on the EPIC cohort. Fruit and vegetable consumption had a protective effect against colorectal, breast, and lung cancer, whereas only fruit had a protective effect against prostate cancer. A higher consumption of fish and lower consumption of red and processed meat were related with a lower risk of colorectal cancer; and higher consumption of fatty fish with lower risk of breast cancer. Calcium and yogurt intake were found to protect against colorectal and prostate cancer. Alcohol consumption increased the risk for colorectal and breast cancer. Finally, adherence to the Mediterranean diet emerged as a protective factor for colorectal and breast cancer. The EPIC study results are in agreement with the latest evidence from leading authorities on cancer prevention and help to inform public prevention policies and strategies.

## 1. Introduction

Worldwide, an estimated 18.1 million new cancer cases (excluding nonmelanoma skin cancer) and nearly 10 million cancer deaths occurred in 2020 [1]. In Europe, the 2020 cancer burden was estimated to have ascended to 2.7 million new cases and 1.3 million deaths [2]. Overall, the burden of cancer incidence and mortality is rapidly growing, and although cancer was once considered to be a disease of high-income countries, it is now one of the most important public health problems worldwide. The increasing cancer burden reflects in part the decline in fertility and increase of life expectancy, but also the economic, societal, and lifestyle changes related to globalization and socioeconomic development [1,3].

Cancer is a disease characterized mainly by loss of genetic control of cell growth and proliferation. Therefore, it is a genetic disease but the factors that originate the disease process are mainly attributable to environmental and lifestyle factors. These include tobacco consumption, diet, excessive body weight and obesity, alcohol consumption, physical inactivity, or sun exposure, among others, meaning there is great potential for cancer prevention [4,5,6]. In relation to modifiable lifestyle factors, there is overwhelming evidence that nutrition, diet, and other related factors such as alcohol intake, physical activity or obesity have an important impact on cancer risk—even more so than smoking—, and that positive behaviour changes can significantly reduce cancer burden [7,8]. However, despite several decades of epidemiological research, the scientific evidence of the effect of many specific foods and nutrients on cancer is still inconsistent or insufficient and attempts to draw solid conclusions have been hampered [5,8,9]. The European Prospective Investigation into Cancer and Nutrition (EPIC), one of the largest cohort studies in the world, was precisely designed to investigate the relationship between diet and cancer and other chronic diseases with the purpose of overcoming previous limitations and contributing to the scientific body of knowledge [10,11].

EPIC is a multi-centre prospective cohort study of 519,978 participants (366,521 women and 153,457 men), most aged 35–70 years, and recruited mostly between 1992 and 1998. Its main aim has been to investigate the relationship of dietary, genetic, lifestyle, and environmental factors with the incidence of cancer and other chronic diseases [5,11]. The study was conducted in 23 centres in 10 different European countries: France, Denmark, Germany, Italy, Greece, The Netherlands, Spain, Norway, Sweden, and the UK. Europe is considered an ideal natural laboratory due to the different dietary habits found in the European population. These include Mediterranean diet patterns typical of southern Italy, Greece, the south of France, and Spain; central European food patterns typical of Germany, the Netherlands, and the north of France; and Nordic diet patterns found in Denmark and Sweden [10]. In addition, the incidence of some major cancers varies significantly between countries and even between regions [10].

Diet over the previous 12 months was assessed at the time of recruitment using validated country-specific questionnaires [12,13]: self-administered semiquantitative food-frequency questionnaires (around 260 food items), semi-quantitative food-frequency questionnaires (combined with dietary record), and diet history questionnaires administered through interviews (with more than 600 food items) [11]. To calibrate the dietary measurement and to correct the errors produced by overestimation or underestimation of food intake, a 24-h recall was performed by a computerized program (EPICSOFT) in a random subsample of 8% of the cohort [14]. In addition, information about lifestyles, habits, and medical history was collected. Weight, height, and waist and hip circumference were assessed, and 385,747 blood samples were collected for hormonal, biochemical, and genetic analyses. Incident cancer cases (above 109,000 cases so far) are regularly identified through automated links with the databases of population cancer registries, except in Germany, France, and Greece, where a combination of methods is used (including monitoring of health insurance records, hospital registries, and active follow-up of participants). Nutrients are analysed using a standardised Food Composition Table (EPIC Nutrient Database ENDB) [15].

Altogether, the EPIC cohort is one of the largest worldwide and its findings regularly contribute to international recommendations on cancer prevention. The last review of the evidence based on EPIC regarding the role of diet in cancer prevention was published more than 10 years ago [9]. Multiple publications with longer follow-up and investigating previously unaddressed diet-related exposures have been published ever since, significantly increasing the contribution of EPIC. Thus, the aim of the current review is to summarize the findings derived from the EPIC study regarding the relationship between diet-related exposures and cancer risk and mortality, considering the four most frequent cancer types in the European population [1]: colorectal, breast, lung, and prostate cancer.

## 2. Materials and Methods

**Figure 1:**
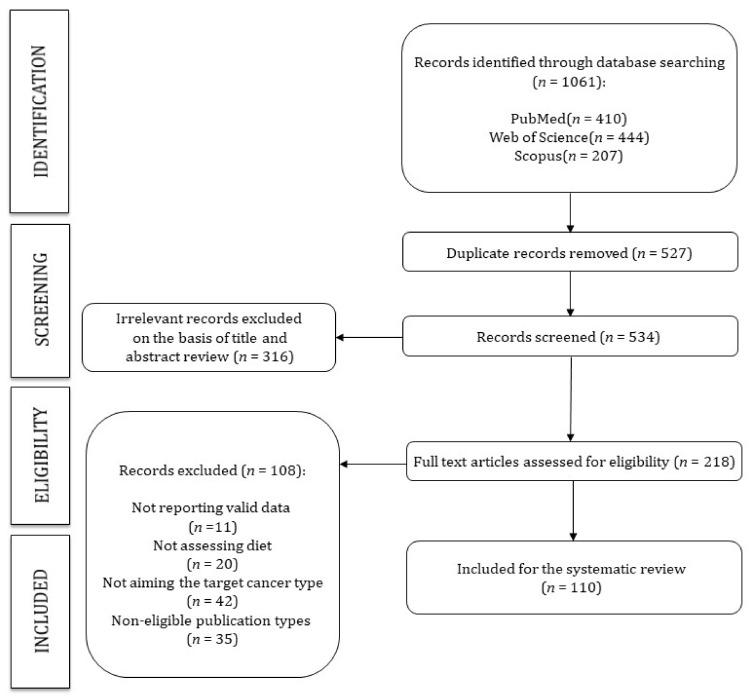
Preferred Reporting Items for Systematic Reviews and Meta-Analyses (PRISMA) flow diagram of study selection, inclusion and exclusion.

This study was reported following the Preferred Reporting Items for Systematic Reviews and Meta-Analyses (PRISMA guidelines) (Figure 1) [16] and was registered in the International Prospective Register of Systematic Reviews PROSPERO (Registration number: CRD42021245178).

### 2.1. Search Strategy

We systematically searched MEDLINE (via PubMed), Scopus, and Web of Science to identify prospective studies from the European Prospective Investigation into Cancer and Nutrition (EPIC) study addressing the effect of diet-related factors on the incidence and mortality of colorectal, breast, lung, and prostate cancer. The search strategy included the following terms: (“European prospective investigation into cancer” OR “European prospective investigation into cancer and nutrition” OR “EPIC study”) AND (“lung cancer” OR “prostate cancer” OR “breast cancer” OR “colorectal cancer” OR “colon cancer” OR “rectal cancer”) AND (“diet” OR “intake” OR “nutrients”). The literature search was performed between February and March 2021 by reviewing citations of the articles considered eligible for the systematic review, and the authors were contacted to obtain missing information when necessary. The complete search strategies used for each database are available in Table S1.

### 2.2. Study Selection

The criteria for including studies were as follows: (i) study design: prospective studies from the EPIC project; (ii) any of the following exposures: food intake/type of diet/nutrient levels/nutritional biomarkers/alcohol consumption; and (iii) outcome: incidence or mortality of colorectal, breast, prostate, or lung cancer. The criteria for excluding studies were as follows: non-eligible publication types, such as review articles, editorials, comments, guidelines, or case reports. No exclusion criteria were applied based on language or journal publication date. The titles, abstracts, and full texts were independently reviewed by 2 reviewers (E.U.-G. and A.C.-L.), and disagreements were solved by consensus or involving a third researcher.

### 2.3. Data Extration and Quality Assessment

The following data was extracted from the original reports: (1) first author and year of publication; (2) tumour site; (3) no. of cases; (4) no. of non-cases; (5) mean follow-up; and (6) main results. The Mendeley software was used for data management.

The Joanna Briggs Institute Critical Appraisal Tool for Systematic Reviews (Table S2) [17] was used to evaluate the risk of bias for cohort studies as in previous cancer and diet reviews [18,19,20]. The assessed methodological criteria included 11 items, each of them with four possible answers: “yes” (criterion met), “no” (criterion not met), “unclear”, and “not applicable” (N/A). A study was considered as “high quality” when the quality score was at least 0.75 (i.e., 75%), whereas studies were considered as “low quality” when the quality score was lower than 0.75. In addition, a score for each criterion was calculated, by dividing the number of positively scored by the total number of included studies, to provide an overview of how well the current literature scores on each criterion.

Data extraction and quality assessment were independently performed by two researchers, and inconsistencies were solved by consensus or involving a third researcher.

### 2.4. Data Synthesis

Due to the overlap of participants among studies, a quantitative meta-analysis was not undertaken. Therefore, the evidence was summarized qualitatively for each of the included cancer types (colorectal, breast, lung, and prostate cancer). The results are shown by type of cancer and grouped according to exposure type following the “Third Expert Report of Diet, Nutrition, Physical Activity and Cancer: a Global Perspective, from World Cancer Research Fund” [9].

## 3. Results

### 3.1. Study Quality

All 110 studies that entered the review (see Figure 1) were high quality studies according to the assessment tool for Systematic Reviews from The Joanna Briggs Institute [17]. Table S3 shows the percentage of studies meeting the quality criteria and provides detailed information on the quality score of each study.

When the studies were analysed by individual domains, 100% of the studies measured the exposure and the outcomes in a valid and reliable manner, identified the potential confounders and took them into account within the study design or in the data analysis, the participants were free of the outcomes of interest at the start of the study, the follow up was completed by a large percentage of participants, and the statistical analysis used was appropriate. It should be taken into account that, among the studies that had two groups, 44.7% had similar groups that were recruited from the same population, and 100% of those studies measured the exposures similarly in order to assign people to the exposed or unexposed groups. Most of the studies presented an appropriate length of time for follow up (93.6%). In no study was it necessary to apply strategies to address incomplete follow up.

### 3.2. Colorectal Cancer

We identified 43 studies on colorectal cancer (Table 1), with a mean follow-up of 9.2 ± 4.0 years.

Wholegrains, vegetables and fruit: A higher consumption of fruits and vegetables combined was related to a lower risk of colorectal cancer: HR 0.86 (0.75–1.00) [21], but no consistent associations were observed for separate consumption of fruits and vegetables [21,22]. Similar findings were observed for the association between fibre consumption and colorectal cancer: HR 0.58 (0.41–0.85) [23], HR 0.79 (0.63–0.99) [24], and HR 0.87 (0.79–0.96) per 10 g/day increase in fibre [25]. A protective effect was also observed between intake of nuts/seeds and the risk of colon cancer in women [26]: HR 0.69 (0.50–0.95), with no associations in men.

Meat, fish, dairy products, and preservation/processing of foods: Two studies showed a protective effect of fish intake on colorectal cancer: HR 0.88 (0.80–0.96) comparing the highest vs. lowest quintile [27], and HR 0.69 (0.54–0.88) comparing >80 g/day vs. <10 g/day of intake [28]. The opposite was found with regards to meat consumption as an increase of red and processed meat consumption associated with a higher risk of colorectal cancer: HR 1.55 (1.19–2.02) per 100-g increase [28]. Pre-diagnostic red meat, processed meat or fibre intakes were not associated with colorectal cancer mortality; however, a marginal trend of processed meat was detected [29]. With regards to calcium, dietary calcium intake was associated with a lower colorectal cancer risk: IRR 0.69 (0.50–0.96) [30], and HR 0.95 (0.91–0.99) per 200 mg/day [31]. There was also an association between milk consumption and colorectal cancer risk: HR 0.93 (0.89–0.98) per 200 g/day [31]. Finally, yogurt intake was associated with a lower risk of colorectal cancer: HR 0.65 (0.48–0.89) [32], and HR 0.65 (0.48–0.89) [33].

Diet patterns: Adherence to the Mediterranean diet [33], the Italian Mediterranean Index [34], and the Modified Mediterranean diet score (mMDS) [35] were found to protect against colorectal cancer with HR 0.54 (0.36–0.81), HR 0.50 (0.35–0.71), and HR 0.89 (0.80–0.99), respectively. In contrast, higher scores (lower nutritional quality diet) in the Food Standards Agency nutrient profiling system dietary index (FSA-NPS DI), and higher values in the Dietary inflammatory index (DII) were associated with a higher risk of colorectal cancer: HR 1.11 (1.01–1.22) [36], and HR 1.15 (1.04–1.27) [37], respectively.

Alcoholic and non-alcoholic drinks: Alcohol consumption was a risk factor for colorectal cancer in six studies [38,39,40,41,42,43]. Of note, a HR of 1.74 (1.08–2.80) was found among participants drinking >3 drinks/day versus those drinking <1 drink/day [38]. Alcohol consumption (per 15-g/d increment) was associated with a higher risk of colorectal cancer: HR 1.05 (1.03–1.07) [39] and HR 1.08 (1.04–1.12) [42]. More specifically, the risk of colorectal cancer was higher when drinking beer (HR 1.38, 1.08–1.77) than wine (HR 0.61–1.21) [42,43]. Finally, total soft drink consumption was a risk factor for colorectal cancer mortality (≥1 glass per day vs. <1 glass per month): HR 1.25 (1.07–1.47) [44].

Other dietary exposures: Several vitamins have been associated with colorectal cancer risk. A protective effect between levels of 25-(OH)D (vitamin D) and colorectal cancer risk was found in several publications [30,45,46]. The cancer risk associated with 10% higher level of circulating 25-(OH)D were: HR 0.97 (0.95 to 0.99) for colorectal; HR 0.95 (0.93 to 0.98) for colon; HR 1.00 (0.97 to 1.03) for rectum [30]. Higher plasma concentrations of vitamins B2 and B6 were associated with a lower colorectal cancer risk [47]: RR 0.71 (0.56–0.91), and RR 0.68 (0.53–0.87), respectively. A lower colon cancer risk was associated with a higher plasma retinol concentration and dietary b-carotene: IRR 0.63 (0.46–0.87), and OR 0.69 (0.52–0.94), respectively [48]. Dietary vitamin C and dietary vitamin E also protected against distal colon cancer (highest vs. lowest quartile): OR 0.60 (0.39–0.93), and OR 0.65 (0.42–0.99), respectively [48]. Finally, associations with colorectal cancer were also found for other variables such as plasma total alkyl-resorcinols [49], plasma methionine [50], plasma choline [50], fatty acids [51], glycaemic index [33], and concentration of zinc [52], among others.

**Table 1:** Evidence synthesis of the association between diet and colorectal cancer in the EPIC study.

Wholegrains, Vegetables, and Fruit			
No. of Cases (Non-Cases)	Mean Follow-Up (Years)	Results, Relative Risk (95% Confidence Interval (CI))	Reference
1329 (476,711)	5.1	The data showed no association between higher intake of nuts and seeds and risk of colorectal, colon, and rectal cancers in men and women combined, but a significant inverse association was observed in subgroup analyses for colon cancer in women at the highest (>6.2 g/d) vs. the lowest: HR 0.69 (0.50–0.95) category of intake, and for the linear effect of log-transformed intake: HR 0.89 (0.80–0.98), with no associations in men.	Jenab 2004 [26]
1721 (518,257)	6.2	The association between fiber and colorectal cancer was significant: HR 0.79 (0.63–0.99)	Bingham 2005 [24]
2819 (449,936)	8.8	Combined consumption of fruit and vegetables was inversely associated with colorectal cancer risk (highest vs. lowest EPIC-wide quintile of consumption): HR 0.86 (0.75–1.00), P trend = 0.04. No association between fruit or vegetable consumption was observed.	van Duijnhoven 2009 [21]
4517 (472,795)	11.0	Total dietary fibre: HR 0.87 (0.79–0.96) per 10 g/day increase in fibre.	Murphy 2012 [25]
3370 (518,078)	13.0	A lower risk of colon cancer was observed with higher self-reported consumption of fruit and vegetables combined (highest vs. lowest quartile): HR 0.87 (0.75–1.01) P trend = 0.02, but no consistent association was observed for separate consumption of fruits and vegetables. Variety in consumption of fruits and vegetables was not associated with a lower risk of colon or rectal cancer.	Leenders 2015 [22]
		**Meat, Fish, Dairy Products, and Preservation/Processing of Foods**	
**No. of Cases** **(Non-Cases)**	**Mean Follow-Up (YEARS)**	**Results, Relative Risk (95% Confidence Interval (CI))**	**Reference**
1329 (476,711)	4.8	Intake of red and processed meat: HR 1.35 (0.96–1.88) highest vs. lowest intake; P trend = 0.03 Red and processed meat calibrated: HR 1.55 (1.19–2.02); P trend = 0.001 per 100-g increase. Intake of fish (>80 g/day vs. <10 g/day): HR 0.69 (0.54–0.88); P trend < 0.001. Fish calibrated: HR 0.46 (0.27–0.77) P trend = 0.003 per 100-g increase.	Norat 2005 [28]
1248 (518,752)	3.9	Greater dietary intake of calcium was associated with a lower colorectal cancer risk: IRR 0.69 (0.50–0.96).	Jenab 2010 [30]
861 (25,639)	NR	There was an interaction between red and processed meat intake and MGMT Ile143Val polymorphism on colorectal cancer risk (P interaction = 0.04): For individuals who carried the variant genotype with higher red and processed meat intake (above median) risk was increased: OR 1.43 (0.82–2.48), compared with those with the common genotype and lower red and processed meat intake (below median). Amongst the common genotype group with higher red and processed meat intake suggested an inverse association: OR 0.75 (0.55–1.01).	Loh 2010 [53]
2050 (23,490)	11.0	Dietary calcium intake was inversely but not statistically significantly associated with colorectal cancer mortality: HR for per 100 mg increase in intake 0.95 (0.88–1.02).	Li 2011 [54]
289 (44,952)	12.0	Yogurt intake: HR 0.65 (0.48–0.89) highest vs. lowest tertile.	Pala 2011 [32]
4513 (472,609)	11.0	Total milk consumption: HR 0.93 (0.89–0.98) per 200 g/day Whole-fat milk: HR 0.90 (0.82–0.99) per 200 g/day Skimmed milk: HR 0.90 (0.79–1.02) per 200 g/day Dietary calcium: HR 0.95 (0.91–0.99) per 200 mg/day; no association observed for non-dairy calcium sources (HR 1.00, 0.81–1.24) per 200 mg/day.	Murphy 2013 [31]
421 (46,297)	11.0	Consumption of yogurt (highest vs. lowest tertile): RR 0.65 (0.48–0.89) P trend = 0.002	Sieri 2015 [33]
3789 (516,189)	4.1	Pre-diagnostic red meat, processed meat or fibre intakes (defined as quartiles and continuous grams per day) were not associated with colorectal cancer mortality among colorectal cancer survivors; however, a marginal trend across quartiles of processed meat was detected (P = 0.053).	Ward 2016 [29]
6291 (460,869)	14.9	Inversely associated with colorectal cancer incidence (highest vs. lowest quintile): Total fish: HR 0.88 (0.80–0.96) P trend = 0.005 Fatty fish: HR 0.90 (0.82–0.98) P trend = 0.009 Lean fish: HR 0.91 (0.83–1.00) P trend = 0.016 Total n-3 LC-PUFA: HR 0.86 (0.78–0.95) P trend = 0.010 Associated with increased risk of colorectal cancer (highest vs. lowest quintile): Dietary ratio of n-6:n-3 LC-PUFA: HR 1.31 (1.18–1.45) P trend < 0.001	Aglago 2020 [27]
1069 (469,869)	6.4	Subjects with higher concentrations of red blood cell stearic acid were at higher risk for colorectal cancer (per 1 mol%): OR 1.23 (1.07–1.42). Conversely, colorectal cancer incidence decreased with increasing proportions of red blood cell n-3 PUFA, particularly eicosapentaenoic acid (per 1 mol%): OR 0.75 (0.62–0.92).	Linseisen 2021 [55]
		**Dietary Patterns**	
**No. of Cases** **(Non-Cases)**	**Mean Follow-Up (Years)**	**Results, Relative Risk (95% Confidence Interval (CI))**	**Reference**
172 (99,828)	6.3	The meat-eaters pattern (meat, poultry, and margarine) was positively associated with colorectal cancer risk (highest vs. lowest quintile): RR 1.58 (0.98–2.53) P trend = 0.02	Kesse 2006 [56]
290 (63,260)	NR	For colorectal cancer in vegetarians compared with meat eaters: IRR 1.39 (1.01–1.91). Comparing vegetarians with nonvegetarians, the risk of colorectal cancer was significantly higher among vegetarians: IRR 1.49 (1.09–2.03).	Key 2009 [57]
435 (44,840)	11.28	The Italian Mediterranean Index was inversely associated with colorectal cancer risk (highest vs. lowest category): HR 0.50 (0.35–0.71) P trend = 0.043. Highest Italian Mediterranean Index score was also significantly associated with reduced risks of any colon cancer: HR 0.54 (0.36–0.81), distal colon cancer: HR 0.44 (0.26–0.75), and rectal cancer: HR 0.41 (0.20–0.81), but not of proximal colon cancer.	Agnoli 2013 [34]
4355 (516,975)	11.6	A decreased risk of colorectal cancer was estimated when comparing the highest (scores 6–9) with the lowest (scores 0–3) adherence to the Centre-Specific Modified Mediterranean diet score and the Modified Mediterranean diet score: HR 0.92 (0.84–1.00) and HR 0.89 (0.80–0.99), respectively. A 2-unit increment in either Mediterranean scale was associated with a borderline statistically significant reduction in colorectal cancer risk (for the Modified Mediterranean diet score): HR 0.96 (0.92–1.00)	Bamia 2013 [35]
421 (46,297)	11.0	Adherence to Mediterranean diet (highest vs. lowest quartile): RR 0.50 (0.35–0.71) P trend = 0.043	Sieri 2015 [33]
5806 (421,701)	15.3	A higher Food Standards Agency nutrient profiling system dietary index (FSAm-NPS DI) score (lower nutritional quality diet) was associated with a higher risk of colorectal cancer (highest fifth vs. lowest quintile): HR 1.11 (1.01–1.22) P trend = 0.02	Deschasaux 2018 [36]
5991(470,169)	14.0	More proinflammatory diets were related to a higher colorectal cancer risk, particularly for colon cancer: Inflammatory Score of the Diet quartile (highest vs. lowest quintile): HR 1.15 (1.04–1.27) for colorectal cancer risk, HR 1.24 (1.09–1.41) for colon cancer, and HR 0.99 (0.83–1.17) for rectal cancer. Associations were more pronounced in men and not significant in women. The inflammatory profile score of the diet was associated with colorectal cancer risk, particularly colon cancer among men (highest vs. lowest quintile): HR 1.62 (1.31–2.01) for colon cancer overall, and HR 2.11 (1.50–2.97) for colon cancer in men.	Jakszyn 2020 [37]
		**Alcoholic and Non-Alcoholic Drinks**	
**No. of Cases** **(Non-Cases)**	**Mean Follow-Up (Years)**	**Results, Relative Risk (95% Confidence Interval (CI))**	**Reference**
1833 (476,899)	6.2	Lifetime alcohol intake was significantly positively associated to colorectal cancer risk (for 15 g/day increase): HR 1.08 (1.04–1.12). Baseline alcohol was significantly positively associated to colorectal cancer risk (for 15 g/day increase): HR 1.09 (1.05–1.13). The colorectal cancer risk for beer (HR 1.38, 1.08–1.77) was higher than wine (HR 1.21, 1.02–1.44).	Ferrari 2007 [42]
407 (23,837)	11.0	Total alcohol consumption: HR 0.70 (0.44–1.13) for alcohol consumption of ≥21 units/week compared with non-drinkers; P trend = 0.14 (not associated with colorectal cancer). Daily consumption of ≥1 unit of wine: HR 0.61 (0.40–0.94) P trend = 0.04	Park 2009 [43]
1367 (NR)	3.6	Among individuals drinking <30 g alcohol/day (highest vs. lowest quintile of folate status): For males: RR 0.79 (0.52–1.23) P trend = 0.19) For females: RR 0.96 (0.67–1.37) P trend = 0.73) Among those drinking >30 g alcohol/day (highest vs. lowest quintile of folate status): For males: RR 0.91 (0.47–1.75) P trend = 0.87 For females: RR 2.59 (0.53–1.75) P trend = 0.47	Eussen, Vollset, Igland 2010 [41]
3759 (343,478)	12.0	Alcohol consumption: HR 0.87 (0.81–0.94)	Aleksandrova 2014 [40]
NR (521,330)	16.4	Total soft drink consumption was positively associated with colorectal cancer deaths (≥1 glass per day vs. <1 glass per month): HR 1.25 (1.07–1.47) P = 0.0047, with statistically non-significant associations found for sugar-sweetened and artificially sweetened soft drinks.	Mullee 2019 [44]
6291 (515,039)	14.9	Greater alcohol consumption was associated with an increased risk of colorectal cancer (per 15-g/day increment): HR 1.05 (1.03–1.07).	Murphy 2019 [39]
154 (45,339)	14.0	An increase in rectal cancer risk among subjects drinking more than 3 drinks/day of alcohol compared with drinkers of less than 1 drink/day of alcohol: HR 1.74 (1.08–2.80)	Bendinelli 2020 [38]
		**Other Dietary Exposures**	
**No. of Cases** **(Non-Cases)**	**Mean Follow-Up (Years)**	**Results, Relative Risk (95% Confidence Interval (CI))**	**Reference**
1078 (518,922)	3.7	Serum C-peptide concentration was positively associated with an increased colorectal cancer risk (highest vs. lowest quintile): OR 1.37 (1.00–1.88) P trend = 0.10 The cancer risk was stronger for colon: OR 1.67 (1.14–2.46) P trend < 0.01; than for rectal cancer: OR 1.42 (0.90–2.25) P trend = 0.35	Jenab 2007 [58]
1365 (NR)	3.6	The relative risks comparing highest to lowest quintile were: Vitamin B2: RR 0.71 (0.56–0.91) P trend = 0.02 Vitamin B6: RR 0.68 (0.53–0.87) P trend < 0.001 Vitamin B12: RR 1.02 (0.80–1.29) P trend = 0.19	Eussen, Vollset, Hustad 2010 [47]
1367 (NR)	3.6	Folate status (highest vs. lowest quintile): RR 0.94 (0.74–1.20) P trend = 0.44 Individuals living in Northern European countries showed an inverse association between plasma folate and rectal cancer risk (highest vs. lowest folate concentrations): RR 0.56 (0.29–1.09) P trend = 0.04	Eussen, Vollset, Igland 2010 [41]
1248 (518,752)	3.9	The cancer risks associated with 10% higher level of circulating 25-(OH)D were: colorectal HR 0.97 (0.95 to 0.99); colon HR 0.95 (0.93 to 0.98); rectum HR 1.00 (0.97 to 1.03). Lower levels of concentration of 25-(OH)D were associated with higher colorectal cancer risk: <25.0 nmol/L: IRR 1.32 (0.87–2.01); and 25.0–49.9 nmol/L: IRR 1.28 (1.05–1.56) Higher concentrations of 25-(OH)D were associated with lower risk: 75.0–99.9 nmol/L: IRR 0.88 (0.68–1.13); and ≥100.0 nmol/L: 0.77 (0.56–1.06)	Jenab 2010 [30]
861 (25,639)	NR	Individuals who carried the variant genotype with higher vitamin E intake had a lower OR of 0.46 (0.26–0.82) whereas those with lower vitamin E intake had an OR of 1.46 (0.98–2.18) compared with those with the common genotype and lower vitamin E intake (P interaction = 0.009). Similarly, the variant genotype group with higher intake of carotene had an inverse association for colorectal cancer in contrast to the common genotype group, with lower carotene intake: OR 0.39 (0.21–0.71) P interaction = 0.005	Loh 2010 [53]
221 (886)	9	Phyto-oestrogen intake not associated with colorectal cancer among men. Among women: Enterolactone intake: OR 0.33 (0.14–0.74); P trend = 0.008 Total enterolignans intake: OR 0.32 (0.13–0.79); P trend = 0.013 Secoisolariciresinol intake: OR 1.60 (0.96–2.69); P trend = 0.074	Ward 2010 [59]
1202 (518,798)	6.1	Participants with 25(OH)D levels in the highest quintile had an HR of 0.69 (0.50–0.93) for colorectal cancer mortality.	Fedirko 2012 [46]
1372 (384,375)	12.0	Incidence rate ratio of distal colon cancer of plasma total alkylresorcinols (highest vs. lowest quartile): IRR 0.48 (0.28–0.83).	Kyrø 2014 [49]
1399 (520,049)	4.5	An association was observed between higher prediagnostic plasma retinol concentration and a lower risk of colon cancer (highest vs. lowest quartile): IRR 0.63 (0.46–0.87) P trend = 0.01. Dietary b-carotene showed an inverse association with colon cancer (highest vs. lowest quartile): OR 0.69 (0.52–0.94) P trend = 0.02. Dietary vitamin C was inversely associated with risk of distal colon cancer (highest vs. lowest quartile): OR 0.60 (0.39–0.93) P trend = 0.02. Dietary vitamin E showed an inverse association with risk of distal colon cancer (highest vs. lowest quartile): OR 0.65 (0.42–0.99) P trend = 0.04.	Leenders 2014 [48]
1367 (NR)	3.7	Plasma methionine: OR 0.79 (0.63–0.99); P trend = 0.05 Plasma choline: OR 0.77 (0.60–0.99); P trend = 0.07 Plasma betaine: OR 0.85 (0.66–1.09); P trend = 0.06	Nitter 2014 [50]
966 (520,482)	3.9	Higher selenium concentrations were associated with a non-significant lower colorectal cancer risk (per 25 lg/L increase): IRR 0.92 (0.82–1.03)	Hughes 2015 [60]
421 (46,297)	11.0	Glycemic index (highest vs. lowest category): RR 1.35 (1.03–1.78) P trend = 0.031	Sieri 2015 [33]
434 (44,758)	11.28	No significant association between dietary total antioxidant capacity and colorectal cancer: HR 0.88 (0.65–1.19) highest category vs. lowest; P trend = 0.353 Dietary total antioxidant capacity in colon cancer (highest vs. lowest tertile): HR 0.63 (0.44–0.89) P trend = 0.008 Dietary total antioxidant capacity in rectal cancer (highest vs. lowest tertile): HR 2.48 (1.32–4.66) P trend = 0.007 Intakes of vitamin C, vitamin E, and ß-carotene not significantly associated with colorectal cancer risk.	Vece 2015 [61]
4517 (472,795)	11.3	Nutrient pattern characterised by high intakes of vitamins and minerals: HR 0.94 (0.92–0.98) per 1 SD. Pattern characterised by total protein, riboflavin, phosphorus and calcium: HR 0.96 (0.93–0.99) per 1 SD. The remaining two patterns were not significantly associated with colorectal cancer risk.	Moskal 2016 [62]
966 (NR)	NR	Circulating concentration of copper (highest vs. lowest quintile): OR 1.50 (1.06–2.13) P trend = 0.02 Circulating concentration of zinc (highest vs. lowest quintile): OR 0.65 (0.43–0.97) P trend = 0.07 Ratio of copper/zinc (highest vs. lowest quintile): OR 1.70 (1.20–2.40) P trend = 0.0005	Stepien 2017 [52]
4517 (472,795)	11	No association between total flavonoid intake and the risk of overall colorectal cancer or any subtype. Total dietary flavonoid intake (highest vs. lowest quintile): HR 1.05 (0.93–1.18) P trend = 0.58 No association with any intake of individual flavonoid subclasses.	Zamora-Ros 2017 [63]
5991 (470,169)	13.9	Total dietary polyphenol intake (as a continuous variable) in women: HR 1.06 (0.99–1.14) Total dietary polyphenol intake (as a continuous variable) in men: HR 0.97 (0.90–1.11)	Zamora-Ros 2018 [64]
1043 (518,957)	8.3	Results for colorectal cancer mortality associated with deficient relative to sufficient 25(OH)D concentrations were: HR 2.24 (1.44–3.49) among cases with the vitamin D-binding protein isoform. HR 0.94 (0.68–1.22) among cases without vitamin D-binding protein.	Gibbs 2020 [45]
1608 (NR)	7.7	Fatty acids: OR 0.51 (0.29–0.90) per unit increase Endogenous metabolites: OR 0.62 (0.50–0.78) per unit change.	Rothwell 2020 [51]

HR: hazard ratio; IRR: incidence rate ratio; NR: not reported; OR: odds ratio; RR: risk ratio.

### 3.3. Breast Cancer

The number of included studies on breast cancer was 41 (Table 2), with a mean follow-up (years) of 10.0 ± 3.0.

Wholegrains, vegetables and fruit: Low consumption of fruit and vegetables combined was associated with a higher risk of breast cancer: HR of 1.76 (1.10–2.82) [65]. The isolated intake of vegetables was also associated with a lower risk (highest vs. lowest quintile): HR 0.87(0.80–0.94) [66], and HR 0.65 (0.53–0.81) [67]. According to subtypes of vegetables, protective effects were found (highest vs. lowest quintile) [67]: HR 0.70 (0.57–0.86) for leafy vegetables; HR 0.75 (0.60–0.94) for fruiting vegetables; and HR 0.82 (0.66–1.01) for raw tomatoes. Conversely, a study did not show associations between vegetable or fruit intake and breast cancer risk [68]. Higher intakes of total dietary fibre and fibre from vegetables were also associated with a lower risk of breast cancer: HR 0.95 (0.89–1.01), and HR 0.90 (0.84–0.96), respectively; but not with fibre from fruit, cereals, or legumes [69].

Meat, fish, dairy products, and preservation/processing of foods: There were no associations with the intake of fish [70], meat intake [53,71], and calcium intake [72]. An association was only shown between high risk of breast cancer and low consumption of fatty fish: HR 1.80 (1.17–2.78) [65].

Diet patterns: The relationship between breast cancer and dietary profiles was also studied [36,73,74,75]. The Mediterranean diet was found to protect against breast cancer (highest vs. lowest score): HR 0.94 (0.88–1.00) [74]. In contrast, a higher score in the FSA-NPS DI was associated with a higher risk of postmenopausal breast cancer (highest vs. lowest quintile): HR 1.08 (1.00–1.16) [36].

Alcoholic and non-alcoholic drinks: The risk of breast cancer increases with alcohol intake: HR 1.05 (1.03–1.07) [76], HR 1.04 (1.03–1.06) [77], HR 1.74 (1.14–2.68) [65] and TE 1.17(1.01–1.35) [78], among others. Particularly, in the consumption of beer/cider and wine (for 1 SD increment), the risk of breast cancer was HR 1.05 (1.03–1.06) and HR 1.04 (1.02–1.06), respectively [76]. Other drinks showed protective effects on breast cancer. Caffeinated coffee intake was associated with lower risk of postmenopausal breast cancer (highest vs. lowest consumption): HR 0.90 (0.82–0.98) [79]. For every 100 ml increase in caffeinated coffee intake, the risk of ER-PR- breast cancer was lower: HR 0.96 (0.93–1.00) [79]. Tea intake [79] and total, sugar-sweetened, and artificially sweetened soft drink consumption [44] were not associated with breast cancer.

Other dietary exposures: Diet rich in β-carotene, riboflavin, thiamine, folate, fibre, iron, calcium, magnesium, potassium, phosphorus, vitamins C and vitamin B6 was associated with a lower risk of breast cancer [80]: HR 0.89 (0.83–0.95). A-carotene and b-carotene had protective effects on oestrogen-receptor–negative breast tumours risk (highest vs. lowest quintile) [81]: OR 0.61 (0.39–0.98), and OR 0.41 (0.26–0.65), respectively. Regarding the relationship between breast cancer and fatty acids, the risk of breast cancer increased with increasing dietary total industrial trans fatty acids intake [82] and levels of total trans-MUFAs [83]: HR 1.14 (1.06–1.23), and OR 1.75 (1.08–2.83), respectively. Specifically with the levels of trans-palmitoleic acid [83], elaidic acid [82], and conjugated linoleic acid [82]: OR 2.24 (1.30–3.86), HR 1.14 (1.06–1.23), and HR 1.11 (1.03–1.20), respectively. Palmitoleic acid was associated: OR 1.37 (1.14–1.64) [84], and HR 1.08 (1.01–1.16) [82]. In addition, a higher dietary glycaemic load was associated with a higher risk factor of breast cancer: HR 1.36 (1.02–1.82) [85], and RR 1.45 (1.06–1.99) [33,86].

**Table 2:** Evidence synthesis of the association between diet and breast cancer in the EPIC study.

Wholegrains, Vegetables, and Fruit			
No. of Cases (Non-Cases)	Mean Follow-Up (Years)	Results, Relative Risk (95% Confidence Interval (CI))	Reference
3659 (281,867)	5.4	No significant associations between vegetable or fruit intake and breast cancer risk (highest vs. lowest quintile): Total vegetable intake: RR 0.98 (0.84–1.14) P trend = 0.65 Total fruit intake: RR 1.09 (0.94–1.25) P trend = 0.11 Fruit and vegetable juices intake: RR 1.05 (0.92–1.20) P trend = 0.51	van Gils 2005 [68]
1355 (33,116)	7.0	There was a statistically higher risk of breast cancer with: Low consumption of fruit and vegetables: HR 1.76 (1.10–2.82)	Engeset 2009 [65]
3747 (110,757)	9.5	Grapefruit intake (≥60 g/day vs. none intake): HR 0.93 (0.77–1.13) P trend = 0.5	Spencer 2009 [87]
1256 (62,699)	9.0	Olive oil intake (highest vs. lowest tertile of g/day/2000 kcal): HR 1.10 (0.92–1.31). There was no association between olive oil and risk of oestrogen or progesterone receptor-positive tumors, but a suggestion of a negative association with oestrogen and progesterone receptor-negative tumors.	Buckland 2012 [88]
1072 (30,438)	11.25	Inverse association between consumption of all vegetables and breast cancer risk (highest vs. lowest quintile): Vegetables (all types): HR 0.65 (0.53–0.81) P trend = 0.003 According to subtypes of vegetables, inverse association between increasing consumption of leafy vegetables, fruiting vegetables and raw tomatoes (highest vs. lowest quintile): Leafy vegetables all types: HR 0.70 (0.57–0.86) P trend = 0.0001 Fruiting vegetables: HR 0.75 (0.60–0.94) P trend = 0.01 Raw tomatoes: HR 0.82 (0.66–1.01) P = trend 0.03 No association between fruit, overall or by subtypes, and breast cancer risk.	Masala 2012 [67]
11,576 (323,273)	11.5	Breast cancer risk was inversely associated with intakes of total dietary fiber (highest vs. lowest quintile): HR 0.95 (0.89–1.01) P trend = 0.03, and fiber from vegetables: HR 0.90 (0.84–0.96) P trend = 0.01, but not with fiber from fruit, cereals, or legumes. For vegetable fiber, stronger associations were observed for oestrogen-receptor–negative and progesterone-receptor–negative (highest vs. lowest quintile): HR 0.74 (0.59–0.93) P trend = 0.01, than for oestrogen-receptor–positive and progesterone-receptor–positive tumors: HR 0.92 (0.81–1.03) P trend = 0.05	Ferrari 2013 [69]
879 (46,297)	11.0	Consumption of vegetables (highest vs. lowest quintile): RR 0.65 (0.53–0.81) P trend = 0.003	Sieri 2015 [33]
10,197 (324,857)	11.5	Vegetable intake was associated with a lower risk of breast cancer (highest vs. lowest quintile): HR 0.87 (0.80–0.94). Although the inverse association was most apparent for oestrogen and progesterone receptor–negative breast cancer (highest vs. lowest quintile): HR 0.74 (0.57–0.96) P trend = 0.03, and oestrogen and progesterone receptor–positive breast cancer: HR 0.91(0.79–1.05) P trend = 0.14. Fruit intake was not significantly associated with total and hormone receptor-defined breast cancer risk.	Emaus 2016 [66]
10,979 (261,119)	15.0	Intakes of fibre (for a 1 SD increment in intake): HR 0.96 (0.94–0.98) Intakes of apple/pear (for a 1 SD increment in intake): HR 0.96 (0.94–0.99) Intakes of carbohydrates (for a 1 SD increment in intake): HR 0.96 (0.95–0.98)	Heath 2020 [76]
**Meat, Fish, Dairy Products, and Preservation/Processing of Foods**			
**No. of Cases** **(Non-Cases)**	**Mean Follow-Up (Years)**	**Results, Relative Risk (95% Confidence Interval (CI))**	**Reference**
4776 (305,895)	6.4	No significant associations between intake of total fish and breast cancer risk were observed (per 10 g fish/day): HR 1.01 (0.99–1.02). When examining lean and fatty fish separately, a positive significant association only in the highest quintile for fatty fish was found (HR 1.13, 1.01–1.26), but test for trend was not significant (*p* = 0.10).	Engeset 2006 [70]
1355 (33,116)	7.0	There was a statistically higher risk of breast cancer with: Low consumption of fatty fish: HR 1.80 (1.17–2.78)	Engeset 2009 [65]
7119 (312,707)	8.8	Processed meat consumption (highest vs. lowest quintile): HR 1.10 (1.00–1.20) P trend = 0.07	Pala 2009 [71]
861 (25,639)	NR	There was no significant joint effect of red and processed meat intake and genotype polymorphism on the risks for breast cancer.	Loh 2010 [53]
7760 (312,225)	8.8	Calcium intake (highest vs. lowest quintile): HR 0.91 (0.83–1.01).	Abbas 2013 [72]
**Dietary Patterns**			
**No. of Cases** **(Non-Cases)**	**Mean Follow-up (years)**	**Results, Relative Risk (95% Confidence Interval (CI))**	**Reference**
585 (37,058)	7.4	Vegetarian population compared to nonvegetarians presented a HR of 0.91 (0.72–1.14) for breast cancer risk (P trend = 0.40)	Travis 2008 [75]
240 (14,567)	9.8	Mediterranean diet score (for 2-point increment): HR 0.88 (0.75–1.03) P trend = 0.12 Mediterranean diet score in premenopausal women (for 2-point increment): HR 1.01 (0.80–1.28) Mediterranean diet score in postmenopausal women (for 2-point increment): HR 0.78 (0.62–0.98)	Trichopoulou 2010 [73]
10,225 (324,837)	11.0	The adapted relative Mediterranean diet was inversely associated with the risk of breast cancer overall and in postmenopausal women (highest vs. lowest score): HR 0.94 (0.88–1.00) P trend = 0.048 and HR 0.93 (0.87–0.99) P trend = 0.037, respectively. The association was more pronounced in oestrogen and progesterone receptor negative tumors: HR 0.80 (0.65–0.99) P trend = 0.043.	Buckland 2013 [74]
12,063 (421,701)	15.3	A higher FSAm-NPS DI score (lower nutritional quality diet) was associated with a higher risk of postmenopausal breast cancer (highest vs. lowest quintile): HR 1.08 (1.00–1.16) P trend = 0.03	Deschasaux 2018 [36]
**Alcoholic and Non-Alcoholic Drinks**			
**No. of Cases** **(Non-Cases)**	**Mean Follow-Up (Years)**	**Results, Relative Risk (95% Confidence Interval (CI))**	**Reference**
4285 (270,403)	6.4	Alcohol intake (per 10 g/d increase): IRR 1.03 (1.01–1.05)	Tjønneland 2007 [89]
1355 (33,116)	7.0	There was a statistically higher risk of breast cancer with: High alcohol consumption: HR: 1.74 (1.14–2.68)	Engeset 2009 [65]
861 (25,639)	NR	There was no evidence of a significant interaction between MGMT Ile143Valpolymorphism and alcohol on breast cancer risk.	Loh 2010 [53]
10,198 (324,862)	11.0	Caffeinated coffee intake was associated with lower risk of postmenopausal breast cancer (high vs. low consumption): HR 0.90 (0.82–0.98) P trend = 0.029. For every 100 ml increase in caffeinated coffee intake, the risk of oestrogen and progesterone receptor–negative breast cancer was lower: HR 0.96 (0.93–1.00). Non-consumers of decaffeinated coffee had lower risk of postmenopausal breast cancer compared to low consumers: HR 0.89 (0.80–0.99) P trend = 0.128. Exclusive decaffeinated coffee consumption was not related to postmenopausal breast cancer risk, compared to any decaffeinated-low caffeinated intake: HR 0.97 (0.82–1.14), or to no intake of any coffee: HR 0.96 (0.82–1.14). Caffeinated and decaffeinated coffee were not associated with premenopausal breast cancer. Tea intake was neither associated with pre- nor post-menopausal breast cancer.	Bhoo-Pathy 2015 [79]
11,576 (323,274)	11.0	Average daily alcohol intake at baseline (per 10 g/day): HR 1.04 (1.03–1.06) *p* value < 0.001 Significant increasing trends were observed between alcohol intake and tumors.	Romieu 2015 [77]
672 (14,338)	14.8	Alcohol consumption higher than 10 g/day: HR 1.30 (1.09–1.54) P trend = 0.007	Masala 2017 [90]
11,576 (323,274)	11.0	For subjects with low intake of fiber (<18.5 g/day), the risk of breast cancer per 10g/day of alcohol intake: HR 1.06 (1.03–1.08).	Romieu 2017 [91]
NR (321,081)	16.4	Total, sugar-sweetened, and artificially sweetened soft drink consumption was not associated with risk of deaths from breast cancer.	Mullee 2019 [44]
430 (359,570)	14.0	Alcohol intake was positively associated with overall breast cancer risk and specifically with oestrogen-receptor-positive tumours with respectively: TE 1.17(1.01–1.35) and TE 1.36(1.08–1.70) for a 1-SD deviation increase of intake.	Assi 2020 [78]
10,979 (261,119)	15.0	Intake of alcohol (for a 1 SD increment in intake): HR 1.05 (1.03–1.07) Intake of beer/cider (for a 1 SD increment in intake): HR 1.05 (1.03–1.06) Intake of wine (for a 1 SD increment in intake): HR 1.04 (1.02–1.06)	Heath 2020 [76]
**Other Dietary Exposures**			
**No. of Cases** **(Non-Cases)**	**Mean Follow-Up (Years)**	**Results, Relative Risk (95% Confidence Interval (CI))**	**Reference**
838 (65,041)	3.4	Fat intake (highest vs. lowest quartile): RR 1.37 (0.99–1.89)	Thiebaut 2001 [92]
280 (15,275)	5.2	Highest intake for isoflavones and lignans (highest vs. lowest quintile): HR 0.98 (0.65–1.48) and HR 0.70 (0.46–1.09), respectively.	Keinan-Boker 2004 [93]
363 (19,571)	7.0	The SIn-7 (the ratio between palmitic acid and palmitoleic acid) was inversely associated with breast cancer risk (highest vs. lowest quintile): OR 0.66 (0.41–1.05) P trend = 0.031 For total trans-MUFAs (highest vs. lowest quintile): OR 1.75 (1.08–2.83) P trend = 0.018. We found an increased risk of breast cancer with increasing levels of trans-palmitoleic acid and elaidic acid: OR 2.24 (1.30–3.86) P trend = 0.0016, and OR 1.45 (0.90–2.33) P trend = 0.12, respectively. We also observed a trend toward increased risk of breast cancer associated with increasing levels of trans-linoleic acid: OR 1.55 (0.91–2.63) P trend = 0.10 No significant association was found with either cis-MUFAs, palmitoleic or oleic acids, or cis -linoleic acid.	Chajès 2008 [83]
7119 (312,707)	8.8	High saturated fat intake (highest vs. lowest quintile): HR 1.13 (1.00–1.27) P trend = 0.038	Sieri 2008 [94]
585 (37,058)	7.4	Moderate isoflavone intake (median intake 10.8 mg/day): HR 1.08 (0.85–1.38) P trend = 0.65 High isoflavone intake (median intake 31.6 mg/day): HR 1.17 (0.79–1.71) P trend = 0.36	Travis 2008 [75]
366 (19,652)	7	No significant associations between breast cancer risk and serum carotenoids, tocopherols and retinol (highest vs. lowest quintile): Serum carotenoids: OR 0.74 (0.47–1.16) P trend = 0.38 Tocopherols: OR 0.68 (0.41–1.10) P trend = 0.26 Retinol: OR 0.85 (0.53–1.35) P trend = 0.34	Maillard 2010 [95]
7502 (338,493)	8.8	Dietary intake of b-carotene, vitamin C and E (highest vs. lowest quintile): In premenopausal women: HR 1.04 (0.85–1.27), HR 1.12 (0.92–1.36) and HR 1.11 (0.84–1.46), respectively. In postmenopausal women: HR 0.93 (0.82–1.04), 0.98 (0.87–1.11) and 0.92 (0.77–1.11) respectively. High intake of b-carotene and vitamin C in postmenopausal women using exogenous hormones (highest vs. lowest quintile): HR 0.79 (0.66–0.96) P trend = 0.06, and HR 0.88 (0.72–1.07) P trend = 0.05, respectively. Overall, dietary intake of b-carotene, vitamin C and E not related to breast cancer risk in neither pre- nor postmenopausal women.	Nagel 2010 [96]
244 (941)	9.0	Phytoestrogen intake not associated with breast cancer among women.	Ward 2010 [59]
11,576 (323,273)	11.5	In postmenopausal women with oestrogen-receptor–negative breast cancer: Glycemic load (highest vs. lowest quintile): HR 1.36 (1.02–1.82) P trend = 0.010 Carbohydate intake (highest vs. lowest quintile): HR 1.41 (1.05–1.89) P trend = 0.009 Further stratification by progesterone receptor status showed slightly stronger associations with ER⁻/PR⁻ breast cancer (highest vs. lowest quintile): HR 1.48 (1.07–2.05) P trend = 0.010 for glycemic load, and HR 1.62 (1.15–2.30) P trend = 0.005 for carbohydrates.	Romieu 2012 [85]
7760 (312,225)	8.8	Vitamin D intake (highest vs. lowest quintile): HR 1.04 (0.94–1.14)	Abbas 2013 [72]
879 (25,187)	11.0	High dietary glycemic load (highest vs. lowest quintile): RR 1.45 (1.06–1.99) P trend = 0.029	Sieri 2013 [86]
11,576 (323,274)	11.5	Total flavonoids intake (highest vs. lowest quintile): HR 0.97 (0.90–1.04) P trend = 0.591 Isoflavones (highest vs. lowest quintile): HR 1.00 (0.91–1.10) P trend = 0.734 Total lignans intake (highest vs. lowest quintile): HR 1.02 (0.93–1.11) P trend = 0.469	Zamora-Ros 2013 [97]
11,575 (356,418)	11.5	An inverse association was observed between dietary folate and breast cancer risk (highest vs. lowest quintile): HR 0.92 (0.83–1.01) P trend = 0.037. In premenopausal women, we observed a statistically significant trend towards lower risk in oestrogen-receptor-negative breast cancer and progesterone-receptor-negative breast cancer (highest vs. lowest quintile): HR 0.66 (0.45–0.96) P trend = 0.042, and HR 0.70 (0.51–0.97) P trend = 0.021, respectively. A reduction in breast cancer risk was observed when comparing the highest with the lowest dietary folate tertiles in women having a high (>12 alcoholic drinks/week) alcohol intake: HR 0.86 (0.75–0.98).	de Batlle 2015 [98]
11,782 (333,376)	6.0	Among postmenopausal women, an intake of lignans was related to a lower risk of dying from breast (highest vs. lowest quartile): HR 0.72 (0.53–0.98). We found no association for other polyphenol classes.	Kyrø 2015 [99]
879 (46,297)	11.0	Glycemic load (highest vs. lowest category): RR 1.45 (1.06–1.99) P trend = 0.029	Sieri 2015 [33]
11,576 (323,274)	11.5	A pattern rich in nutrients found in animal foods loading on cholesterol, protein, retinol, vitamins B12, and D was not associated with BC risk. Diet rich in β-carotene, riboflavin, thiamin, vitamins C and B6, fibre, Fe, Ca, K, Mg, P and folate, was inversely associated with BC risk (highest vs. lowest quintile): HR 0.89 (0.83–0.95) P trend < 0.01	Assi 2016 [80]
1502 (519,966)	13.0	A-carotene and b-carotene were inversely associated with risk of oestrogen-receptor–negative breast tumors (highest vs. lowest quintile): OR 0.61 (0.39–0.98), and OR 0.41 (0.26–0.65), respectively.	Bakker 2016 [81]
2982 (516,120)	11.5	A high level of palmitoleic acid (highest vs. lowest quartile): OR 1.37 (1.14–1.64) P trend = 0.0001 Levels of industrial trans-fatty acids were positively associated with oestrogen-receptor–negative tumors (highest vs. lowest tertile): OR 2.01(1.03–3.90) P trend = 0.047 No significant association was found between n-3 polyunsaturated fatty acids and breast cancer risk, overall or by hormonal receptor.	Chajès 2017 [84]
2491 (365,412)	11.5	Plasma levels of folate and vitamin B12 not significantly associated with the risk of breast cancer. Vitamin B12 status and breast cancer risk in women consuming above the median value of alcohol intake (highest vs. lowest quartile): OR 1.26 (1.00–1.58) P trend = 0.05 Vitamin B12 status with breast cancer risk in women with plasma folate levels below the median value (highest vs. lowest quartile): OR 1.29 (1.02- 1.62) P trend = 0.03	Matejcic 2017 [100]
13,241 (305,366)	8.1	Dietary total industrial trans fatty acids intake (highest vs. lowest quintile): HR 1.14 (1.06–1.23) P trend = 0.001 Elaidic acid intake (highest vs. lowest quintile): HR 1.14 (1.06–1.23) P trend = 0.001 Dietary total ruminant trans fatty acids intake (highest vs. lowest quintile): HR 1.09 (1.01–1.17) P trend = 0.015 Conjugated linoleic acid intake (highest vs. lowest quintile): HR 1.11 (1.03–1.20) P trend = 0.001 Palmitelaidic acid intake: HR 1.08 (1.01–1.16) P trend = 0.028	Matta 2021 [82]

HR: hazard ratio; IRR: incidence rate ratio; NR: not reported; OR: odds ratio; RR: risk ratio; TE: total effects.

### 3.4. Lung Cancer

A total of 11 studies on lung cancer were included (Table 3), with a mean follow-up (years) of 8.7 ± 4.5.

Wholegrains, vegetables and fruit: The combination of eating fruits and vegetables was associated with a lower lung cancer risk (per 100 g/day increase of intake): HR of 0.94 (0.89–0.99) [101]. Fruit consumption also protects against lung cancer risk (highest vs. lowest quintile): HR 0.75 (0.49–0.96) [102], and HR 0.60 (0.46–0.78) [103]. Lung cancer risk decreased with higher vegetable consumption (per 100 g/day increase) in one study: HR 0.92 (0.85–0.99) [102]. Conversely, two studies did not show associations between vegetable consumption and lung cancer [101,103].

Meat, fish, dairy products, and preservation/processing of foods: There were no consistent associations between meat and fish consumption and the risk of lung cancer [104].

Diet patterns: Higher scores in the FSA-NPS DI were associated with a higher lung cancer risk (highest vs. lowest quintile): HR 1.26 (1.06–1.51) [36].

Alcoholic and non-alcoholic drinks: Neither ethanol intake at recruitment nor mean lifetime ethanol intake was associated with lung cancer [105].

Other dietary exposures: A lower risk for lung cancer was observed for elevated serum levels of B6 and for serum methionine (fourth vs. first quartile): OR 0.44 (0.33–0.60), and OR 0.52 (0.39–0.69), respectively [106]. Above-median levels of both were associated with a lower lung cancer risk overall: OR 0.41 (0.31–0.54) [106], as well as separately among never, former and current smokers: OR 0.36 (0.18–0.72), OR 0.51 (0.34–0.76), and OR 0.42 (0.27–0.65), respectively [106]. Finally, vitamin C concentration, vitamin K2 intake and haem iron intake (highest vs. lowest categories) were associated with the risk of lung cancer: HR 0.57 (0.41–0.81) [107], HR 0.38 (0.20–0.71) [108], and HR 1.16 (1.02–1.32) [109], respectively.

**Table 3:** Evidence synthesis of the association between diet and lung cancer in the EPIC study.

Wholegrains, Vegetables, and Fruit			
No. of Cases (Non-Cases)	Mean Follow-Up (Years)	Results, Relative Risk (95% Confidence Interval (CI))	Reference
860 (477,161)	NR	Fruit consumption (highest vs. lowest quintile): HR 0.60 (0.46–0.78) P trend = 0.0099 No association between vegetable consumption or vegetable subtypes and lung cancer risk.	Miller 2004 [103]
1126 (477,464)	6.4	Fruit consumption was significantly inversely associated with lung cancer risk (highest vs. lowest quintile): HR 0.75 (0.49–0.96). Lung cancer risk significantly decreased with higher vegetable consumption (per 100 g/day increase in vegetable intake): HR 0.90 (0.81–0.99) in smokers and HR 0.92 (0.85–0.99) overall.	Linseisen 2007 [102]
1830 (476,705)	8.7	A 100 g/day increase in fruit and vegetables consumption was associated with a reduced lung cancer risk: HR 0.94 (0.89–0.99). A 100 g/day increase in fruit consumption was associated with a reduced lung cancer risk: HR 0.94 (0.88–1.01). A 100 g/day increase in vegetables consumption was associated with a reduced lung cancer risk: HR 0.94 (0.83–1.07).	Büchner 2010 [101]
**Meat, Fish, Dairy Products, and Preservation/Processing of Food**			
**No. of Cases** **(Non-Cases)**	**Mean Follow-Up (Years)**	**Results, Relative risk (95% Confidence Interval (CI))**	**Reference**
1822 (476,199)	8.7	There were no consistent associations between meat consumption and the risk of lung cancer: Red meat (per 50 g intake/day): RR 1.06 (0.89–1.27) Processed meat (per 50 g g/day): RR 1.13 (0.95–1.34) Consumption of white meat and fish were also not associated with the risk of lung cancer.	Linseisen 2011 [104]
**Dietary Patterns**			
**No. of Cases** **(Non-Cases)**	**Mean Follow-Up (Years)**	**Results, Relative Risk (95% Confidence Interval (CI))**	**Reference**
3654 (421,701)	15.3	A higher FSAm-NPS DI score (lower nutritional quality diet) was associated with a higher lung cancer risk in men and women (highest vs. lowest quintile): HR 1.26 (1.06–1.51) P trend = 0.02, and HR 2.33 (1.23–4.43) P trend = 0.008, respectively.	Deschasaux 2018 [36]
**Alcoholic and Non-Alcoholic Drinks**			
**No. of Cases** **(Non-Cases)**	**Mean Follow-Up (Years)**	**Results, Relative Risk (95% Confidence Interval (CI))**	**Reference**
1119 (477,471)	6.4	Neither ethanol intake at recruitment nor mean lifelong ethanol intake significantly associated with lung cancer.	Rohrmann 2006 [105]
**Other Dietary Exposures**			
**No. of Cases** **(Non-Cases)**	**Mean Follow-Up (Years)**	**Results, Relative risk (95% Confidence Interval (CI))**	**Reference**
899 (384,848)	NR	A lower risk for lung cancer was seen for elevated serum levels of B6 and for serum methionine (fourth vs. first quartile): OR 0.44 (0.33–0.60) P trend < 0.000001, and OR 0.52 (0.39–0.69) P for trend <0.000001, respectively. Above-median levels of both were associated with a lower lung cancer risk overall: OR 0.41 (0.31–0.54), as well as separately among never, former and current smokers: OR 0.36 (0.18–0.72), OR 0.51 (0.34–0.76), and OR 0.42 (0.27–0.65), respectively.	Johansson 2010 [106]
128 (22,585)	10.2	Dietary intake of vitamin K2 (highest vs. lowest quintile): HR 0.38 (0.20–0.71) P trend = 0.002	Nimptsch 2010 [108]
892 (1748)	5.2	PA/(pyridoxal þ PLP) levels: OR 1.52 (1.27–1.81) P < 0.001	Zuo 2018 [110]
3914 (15,443)	12.0	Plasma vitamin C concentration (highest vs. lowest quartile): HR 0.57 (0.41–0.81)	Myint 2019 [107]
3731 (413,015)	13.9	Haem iron intake (as a continuous variable): HR 1.03 (1.00–1.07) per 0.3 mg/1000 kcal Haem iron intake (highest vs. lowest quintile): HR 1.16 (1.02–1.32) P trend = 0.035 Non-haem iron intake (as a continuous variable): HR 0.96 (0.92–1.00) per 1.2 g/1000 kcal Non-haem iron intake (highest vs. lowest quintile): HR 0.90 (0.79–1.02) P trend = 0.068 Total iron intake (as a continuous variable): HR 0.98 (0.94–1.02) per 1.3 g/1000 kcal Total iron intake (highest vs. lowest quintile): HR 0.95 (0.84–1.07) P trend = 0.20	Ward 2019 [109]

HR: hazard ratio; NR: not reported; OR: odds ratio; RR: risk ratio.

### 3.5. Prostate Cancer

Twenty-five studies on prostate cancer were selected (Table 4), with a mean follow-up (years) of 9.4 ± 3.8.

Wholegrains, vegetables and fruit: No associations between fruits and vegetables consumption and prostate cancer risk were observed in one study [111]. However, these results were later disconfirmed in another study that showed protective associations with the consumption of fruits (highest vs. lowest quintile): HR 0.91 (0.83–0.99) [112]. Overall, there was no association between dietary fibre intake (total, cereal, fruit or vegetable fibre) and prostate cancer risk [113].

Meat, fish, dairy products, and preservation/processing of foods: There were no associations between prostate cancer and the intake of dairy products, fish, fat from red meat, and dietary fat [114]. However, there was an association between the risk of high-grade prostate cancer and the intakes of total, monounsaturated, and polyunsaturated fats [114]. For each 10% increase in the observed intake of total fat, the risk of high-grade prostate cancer decreased: HR 0.83 (0.72–0.95) [114]. Each 5% increase in monounsaturated and polyunsaturated fat intake was associated with a lower risk of high-grade prostate cancer: HR 0.82 (0.70–0.97) and HR 0.77 (0.62–0.97), respectively [114]. Higher fat intake from dairy products was also associated with lower risk of localized prostate cancer [114]: HR 0.92 (0.86–0.99) and HR 0.90 (0.82–0.99), for the observed and calibrated intakes, respectively. In contrast, yoghurt intake, total dietary calcium intake, calcium intake and protein intake from dairy products were associated with an increased risk of prostate cancer (highest vs. lowest quintile): HR 1.17 (1.04–1.31), HR 1.17 (1.00–1.35), HR 1.18 (1.03–1.36), and HR 1.22 (1.07–1.41), respectively [115].

Diet patterns: One study found a borderline association between a higher score in the FSA-NPS DI and a higher risk of prostate cancer (highest versus lowest quintile): HR 1.07 (0.98–1.17) [36].

Alcoholic and non-alcoholic drinks: Neither alcohol consumption at baseline nor average lifetime alcohol consumption was associated with risk for prostate cancer [116]. No evidence of association for consumption of total, caffeinated or decaffeinated coffee or tea and risk of total prostate cancer or cancer by stage, grade or fatality (highest vs. lowest consumers) [117]. Neither total, sugar-sweetened, and artificially sweetened soft drink consumption was associated with risk of death from prostate cancer [44].

Other dietary exposures: There was a protective effect between menaquinone intake and prostate cancer: HR 0.37 (0.16–0.88) for advanced prostate cancer [118], HR 0.65 (0.44–0.97) for prostate cancer incidence [108], OR 1.38 (1.03–1.86) for serum undercarboxylated osteocalcin (ucOC)/intact osteocalcin (iOC) ratio in advanced-stage prostate cancer [119], and OR 1.21 (1.00–1.46) for serum ucOC/iOC ratio in high-grade prostate cancer [119]. Fatty acids were a risk factor for prostate cancer: HR 1.08 (1.01–1.15) for butyric acid intake [120], HR 1.05 (1.00–1.11) for eicosenoic acid intake [120], HR 1.07 (1.00–1.14) for eicosapentaenoic acid intake [120], and OR 1.27 (1.01–1.60) for plasma phytanic acid concentration [121]. Finally, the serum concentration of insulin-like growth factor I was also a risk factor for prostate cancer [122].

**Table 4:** Evidence synthesis of the association between diet and prostate cancer in the EPIC study.

Wholegrains, Vegetables, and Fruit			
No. of Cases (Non-Cases)	Mean Follow-Up (Years)	Results, Relative Risk (95% Confidence Interval (CI))	Reference
1104 (129,440)	4.8	No significant associations between fruit and vegetable consumption and prostate cancer risk were observed (highest vs. lowest quintile): Total fruits: RR 1.06 (0.84–1.34) Total vegetables: RR 1.00 (0.81–1.22) Total fruits and vegetables combined: RR 1.00 (0.79–1.26)	Key 2004 [111]
2747 (139,843)	8.7	Overall, no association between dietary fiber intake (total, cereal, fruit or vegetable fiber) and prostate cancer risk, although calibrated intakes of total fiber and fruit fiber were associated with non-statistically significant reductions in risk (per 10 g/day): IRR 0.91 (0.81–1.02) P trend = 0.12 and IRR 0.95 (0.89–1.00) P trend = 0.06, respectively.	Suzuki 2009 [113]
7036 (135,200)	13.9	Total fruit intake (highest vs. lowest quintile): HR 0.91 (0.83–0.99) P trend = 0.01 Citrus fruits intake (highest vs. lowest quintile): HR 0.94 (0.86–1.02) P trend = 0.01	Perez-Cornago 2017 [112]
**Meat, Fish, Dairy Products, and Preservation/Processing of Foods**			
**No. of Cases** **(Non-Cases)**	**Mean Follow-Up (Years)**	**Results, Relative Risk (95% Confidence Interval (CI))**	**Reference**
2727 (139,793)	8.7	Yoghurt intake was associated with an increased risk (highest vs. lowest quintile): HR 1.17 (1.04–1.31) P trend = 0.02, but there was no evidence of an association with intakes of milk and milk beverages or cheese. Total protein intake was not positively associated with increased risk (highest vs. lowest quintile): HR 1.17 (0.96–1.44) P trend = 0.07. Protein from dairy foods was significantly associated with an increased risk (highest vs. lowest quintile): HR 1.22 (1.07–1.41) P trend = 0.02. Total dietary calcium intake and calcium intake from dairy foods were also associated with an increased risk (highest vs. lowest quintile): HR 1.17 (1.00–1.35) P trend = 0.01 for total dietary calcium, and HR 1.18 (1.03–1.36) P trend = 0.02 for dairy calcium. Calcium intake from nondairy foods was not associated with risk. An increment of 35 g day^−1^ dairy protein was associated with an HR of 1.32 (1.01–1.72) P trend = 0.04, and increments of 0.3 g day^−1^ of total calcium and dairy calcium were associated with HR of 1.09 (1.01–1.16) P trend = 0.02, and HR 1.07 (1.00–1.14) P trend = 0.04, respectively.	Allen, Key 2008 [115]
2727 (139,793)	8.7	There were no significant associations between prostate cancer risk and fat from red meat, dairy products, and fish. There was a significant inverse relation between fat from dairy products and risk of localized prostate cancer (each 5% increase in energy from dairy fat): HR 0.92 (0.86–0.99) and HR 0.90 (0.82–0.99), for the observed and calibrated intakes, respectively. There was no significant association between dietary fat (total, saturated, monounsaturated, and polyunsaturated fat and the ratio of polyunsaturated to saturated fat) and risk of prostate cancer: Total fat intake (highest vs. lowest quintile): HR 0.96 (0.84–1.09) P trend = 0.155. There was a significant inverse association between the observed and calibrated intakes of total, monounsaturated, and polyunsaturated fats and the risk of high-grade prostate cancer: For each 10% increase in the observed intake of total fat, the risk of high-grade prostate cancer decreased: HR 0.83 (0.72–0.95). Each 5% increase in monounsaturated and polyunsaturated fat intake was associated with a lower risk of high-grade prostate cancer: HR 0.82 (0.70–0.97) and HR 0.77 (0.62–0.97), respectively.	Crowe, Key 2008 [114]
861 (25,639)	NR	There was no significant joint effect of red and processed meat intake and genotype polymorphism on the risks for prostate cancer.	Loh 2010 [53]
**Dietary Patterns**			
**No. of Cases** **(Non-Cases)**	**Mean Follow-Up (Years)**	**Results, Relative Risk (95% Confidence Interval (CI))**	**Reference**
6745 (421,701)	15.3	A higher FSAm-NPS DI score (lower nutritional quality diet) score was associated with a borderline significant higher risk of prostate cancer (highest vs. lowest quintile): HR 1.07 (0.98–1.17) P trend = 0.04	Deschasaux 2018 [36]
**Alcoholic and Non-Alcoholic Drinks**			
**No. of Cases** **(Non-Cases)**	**Mean Follow-Up (Years)**	**Results, Relative Risk (95% Confidence Interval (CI))**	**Reference**
630 (NR)	3.4	Insulin-like growth factor I serum concentration (highest vs. lowest third): OR 1.39 (1.02–0.89)	Allen 2007 [122]
2655 (139,952)	8.7	Neither alcohol consumption at baseline nor average lifetime alcohol consumption was significantly associated with risk for prostate cancer: Alcohol intake: RR 0.88 (0.72–1.08) highest intake (≥60 g per day) vs. lowest (0.1–4.9 g/d) Average lifetime alcohol intake: RR 1.09 (0.86–1.39)	Rohrmann 2008 [116]
861 (25,639)	NR	A higher prostate cancer risk was seen with higher alcohol intake among the MGMTIle143Val polymorphism with the variant genotype compared to the MGMTIle143Val polymorphism with the common genotype with lower alcohol intake: OR 2.08 (1.21–3.57) P interaction = 0.0009	Loh 2010 [53]
NR (521,330)	16.4	Total, sugar-sweetened, and artificially sweetened soft drink consumption was not associated with risk of death from prostate cancer.	Mullee 2019 [44]
7036 (135,160)	14.0	No evidence of association for consumption of total, caffeinated or decaffeinated coffee or tea and risk of total prostate cancer or cancer by stage, grade or fatality (highest vs. lowest consumers): Coffee intake for total prostate cancer: HR 1.02 (0.94–1.09) Tea intake for total prostate cancer: HR 0.98 (0.90–1.07) Coffee intake for total fatal prostate cancer: HR 0.97 (0.79–1.21) Tea intake for total fatal prostate cancer: HR 0.89 (0.70–1.13)	Sen 2019 [117]
**Other Dietary Exposures**			
**No. of Cases** **(Non-Cases)**	**Mean Follow-Up (Years)**	**Results, Relative Risk (95% Confidence Interval (CI))**	**Reference**
966 (136,035)	6.0	None of the micronutrients examined were significantly associated with prostate cancer risk. The risk of advanced disease (highest vs. lowest quintile of plasma concentrations): IRR 0.40 (0.19–0.88) for lycopene and IRR 0.35 (0.17–0.78) for the sum of carotenoids.	Key 2007 [123]
959 (149,041)	11.0	Plasma selenium concentration was not associated with prostate cancer risk (highest vs. lowest quintile): RR 0.96 (0.70–1.31) P trend = 0.25	Allen, Naomi 2008 [124]
962 (152,495)	4.2	An inverse association between palmitic acid and the risk of total, localized, and low-grade prostate cancer (highest vs. lowest quintile): RR 1.47 (0.97–2.23) P trend = 0.032, RR 1.90 (1.03–3.49) P trend = 0.013, and RR 1.93 (1.02–3.64) P trend = 0.045, respectively. An inverse association between stearic acid and the risk of total and localized prostate cancer (highest vs. lowest quintile): RR 0.77 (0.56–1.06) P trend = 0.03, and RR 0.60 (0.38–0.94) P trend = 0.014, respectively. Significant positive associations between myristic, α-linolenic and eicosapentaenoic acids and risk of high-grade prostate cancer: RR 1.79 (0.80–3.98), 1.79 (0.91–3.53), and 2.00 (1.07–3.76), respectively.	Crowe, Allen 2008 [125]
268 (11,660)	8.6	Total menaquinone intake (highest vs. lowest quartile): HR 0.65 (0.39–1.06) Total menaquinone intake for advanced prostate cancer: HR 0.37 (0.16–0.88) P trend = 0.03 Phylloquinone intake (highest vs. lowest quartile): HR 1.02 (0.70–1.48)	Nimptsch 2008 [118]
250 (11,678)	8.0	Serum ucOC/iOC ratio in advanced-stage prostate cancer (per 0.1 increment): OR 1.38 (1.03–1.86) Serum ucOC/iOC ratio in high-grade prostate cancer (per 0.1 increment): OR 1.21 (1.00–1.46)	Nimptsch 2009 [119]
652 (152,805)	4.1	No significant association between 25-hydroxyvitamin D and risk of prostate cancer (highest vs. lowest quintile): OR 1.28 (0.88–1.88) P trend = 0.188	Travis, Crowe 2009 [126]
950 (136,050)	4.2	Plasma concentrations of phyto-oestrogen genistein (highest vs. lowest quintile): RR 0.74 (0.54–1.00) P trend = 0.05	Travis, Spencer 2009 [127]
180 (22,585)	10.2	Dietary intake of menaquinone (highest vs. lowest quintile): HR 0.65 (0.44–0.97) P trend = 0.03	Nimptsch 2010 [108]
566 (152,834)	4.8	Plasma phytanic acid concentration: OR 1.27 (1.01–1.60) P trend = 0.04	Price 2010 [121]
248 (11,680)	NR	Glucosinolate intake (per 10 mg/d increment): OR 0.72 (0.53–0.96)	Steinbrecher 2010 [128]
204 (812)	9	Total enterolignans intake: OR 1.19 (0.77–1.82) P trend = 0.44	Ward 2010 [59]
962 (152,495)	NR	A high score on a factor reflecting a long-chain n23 PUFA pattern (fatty acids) was associated with greater risk of prostate cancer (highest vs. lowest quintile): OR 1.36 (0.99–1.86) P trend = 0.041	Dahm 2012 [129]
4606 (134,399)	10.0	Prostate cancer risk was not associated with intake of nitrosamines (highest vs. lowest quintile): HR 0.91 (0.81- 1.03) for endogenous Nitrosocompounds, and HR 1.04 (0.95–1.18) for N-Nitrosodimethlyamine. There was also no association with heme iron (highest vs. lowest quintile): HR 1.00 (0.88–1.39).	Jakszyn 2012 [130]
5916 (117,082)	14.0	Dry cakes/biscuits and butter intakes for low-grade prostate cancer: HR 1.07 (1.03–1.11) P = 0.01 Dry cakes/biscuits and butter intakes for aggressive prostate cancer: HR 1.08 (1.04–1.13) P = 0.02	Papadimitriou 2020 [131]
7036 (135,203)	13.9	Butyric acid intake calibrated for advanced stage prostate cancer (for 1 SD increase): HR 1.08 (1.01–1.15) P trend = 0.026 Eicosenoic acid intake calibrated for fatal prostate cancer (for 1 SD increase): HR 1.05 (1.00–1.11) P trend = 0.048 Eicosapentaenoic acid intake calibrated for fatal prostate cancer (for 1 SD increase): HR 1.07 (1.00–1.14) P trend = 0.045	Perez-Cornago 2020 [120]

HR: hazard ratio; IRR: incidence rate ratio; NR: not reported; OR: odds ratio; RR: risk ratio.

## 4. Discussion

Colorectal cancer: Several EPIC studies have found protective effects against colorectal cancer risk of the following diet-related factors: consumption of fish, dietary fibre, fruits and vegetables, nuts/seeds, dietary calcium, milk, yogurt, circulating vitamin D concentration, plasma concentrations of vitamins B2 and B6, plasma retinol concentration, dietary b-carotene, dietary vitamin C, and dietary vitamin E. On the contrary, alcohol and red and processed meat consumption were associated with a significant increase in colorectal cancer risk. Adherence to a traditional Mediterranean diet was strongly associated with a reduction of colorectal cancer risk, whereas higher FSA-NPS DI scores, i.e., lower nutritional quality of the diet, and higher DII scores, i.e., a more pro-inflammatory diet, were particularly associated with higher risks of cancer of the colon-rectum. However, the associations for the DII scores were more evident in men and not significant in women. These sex-differences can be due to hormonal influences because oestrogen plays a key protective role in the pathogenesis of colorectal cancer in women [132]. Therefore, diet and hormonal components can affect colorectal cancer risk differently in women than in men [133].

The EPIC study findings are in line with the World Cancer Research Fund/American Institute of Cancer Research (WCRF/AICR) latest report [9] that concluded that there is strong evidence to support that consuming foods containing dairy products, dietary fibre, and dietary calcium decreases the risk of colorectal cancer while consuming alcohol and red and processed meat increases it. According to this report, there is also limited evidence supporting that the intake of fish, carotene, vitamin C, and vitamin D might decrease colorectal cancer risk; however, a recent mendelian randomisation study on vitamin D and risk of seven cancers concluded that there is limited evidence of a linear causal relationship between circulating vitamin D concentration and colorectal cancer risk [134]. Further, ingested red and processed meat is classified following the International Agency for Research on Cancer (IARC) as a probable human carcinogen (group 2A) and carcinogen to humans (group 1), respectively [135]. A current review of 45 meta-analyses also found convincing and biologically plausible evidence for the association between lower risk of colorectal cancer and higher dietary fibre intake, higher dietary calcium and yogurt intake, and lower alcohol and red meat intake [136]. The EPIC study findings appear to be in agreement with the latest systematic review from the WCRF and AICR [8] and a recent umbrella review of meta-analyses of prospective observational studies [136]. Hence, they largely support existing cancer prevention recommendations that advise increased fibre and dairy products intake and limited red meat and alcohol intake to reduce colorectal cancer risk.

Breast cancer: The EPIC results showed that intake of vegetables, fruits and vegetables, total dietary fibre, fibre from vegetables, and fatty fish protect against breast cancer, whereas no associations were found for fruit or fibre derived from fruit, cereals, or legumes. However, one study did not find associations between fruit or vegetable intake and breast cancer risk [68]. Since these results were obtained during the first years of EPIC, the relatively short follow-up might not best reflect the true association. Later, significant associations were found in longer follow-up studies [33,65,66,67]. Lower breast cancer risk was associated with adherence to a Mediterranean diet whereas diets with lower nutritional quality were associated with a higher risk of postmenopausal breast cancer. Overall, stronger associations were found for progesterone-receptor–negative and oestrogen-receptor–negative cancer types for vegetable intake, fibre from vegetables, and Mediterranean diet. Conversely, alcohol intake clearly and strongly increased the risk of breast cancer regardless of the menopausal status. A diet rich in β-carotene, riboflavin, thiamine, folate, fibre, iron, calcium, magnesium, potassium, phosphorus, vitamins C and vitamin B6 protects against breast cancer. In addition, high saturated fatty acids intake, and high dietary glycaemic load were all significantly associated with a higher risk of breast cancer.

The EPIC study findings are concordant with the WCRF/AICR third expert report [9] that concluded that there is strong evidence that consuming alcoholic drinks increases the risk of breast cancer both in pre- and postmenopausal women. According to the report, there is also some evidence that the consumption of non-starchy vegetables may reduce the risk of oestrogen-receptor–negative breast cancer; the consumption of foods containing carotenoids the risk of breast cancer in general; and the consumption of dairy products and diets high in calcium the risk of premenopausal breast cancer. The evidence available for the risk of postmenopausal breast cancer is more limited but points in the same direction. As seen in the results from the WCRF and AICR [9] and the EPIC study, the role of dietary factors in the development of breast cancer is not entirely resolved. Alcohol is the only established dietary risk factor, probably acting by increasing levels of endogenous oestrogen and other hormones associated with this cancer [137,138,139,140]. Findings from a recent systematic review of dietary patterns and breast cancer risk suggest that patterns including vegetables, red and processed meats, and that limit saturated fat may reduce breast cancer risk [141]. The association between saturated fat and breast cancer has been very controversial over the past years, with some studies such as EPIC supporting a positive relationship [142,143] and others not finding enough evidence of a relationship [144,145]. More studies are needed to confirm these results and to identify the biological pathways that may be the reason for the association.

Lung cancer: The effect of diet on lung cancer has been investigated less in the EPIC study, with only 11 studies reported since 2004. Results from EPIC overall have shown that intake of fruit and vegetables protects against lung cancer. No consistent associations were found for meat and fish consumption. A lower nutritional quality of diet was associated with a higher lung cancer risk in men and women. On the other hand, a decreased risk of lung cancer was seen for high serum levels of B6 and methionine, even among former and current smokers. Along the same lines, dietary intake of vitamin C, vitamin K2 and haem iron were also associated with a lower risk of developing lung cancer.

The EPIC results regarding vitamin C and fruit and vegetables intake are consistent with the epidemiological evidence moderately supporting a decrease in lung cancer risk [9,146,147,148]. The WCRF and AICR report also concluded that food and fruits containing retinol, β-carotene or carotenoids probably decrease the risk of lung cancer, while there is strong evidence that taking high-dose β-carotene supplements increases the risk in current and former smokers. Vegetable intake might also have a protective role in current and former smokers, but more evidence is needed, as well as for red and processed meat, alcoholic drinks, and isoflavones intake. The latest WRCF/AICR report also highlights the robustly convincing role that drinking water containing arsenic has in increasing the risk of lung cancer [9]. Unquestionably, more studies are warranted to explore these results, but since smoking is the main cause of lung cancer, the most effective measure of prevention among smokers remains to stop smoking.

Prostate cancer: EPIC studies regarding prostate cancer and its relationship with diet have yielded controversial results. One study showed protective associations between consumption of fruits and prostate cancer [112], but no associations were observed with fruits or vegetables consumption in another study [111]. This may be also due to the relatively short follow-up in this later study. High total dietary calcium, intake of yoghurt, and calcium and protein from dairy products were significant associated with a moderate increase in risk of prostate cancer. However, there was no evidence of association with the intake of dairy products. Results associated with diets rich in calcium are consistent with scientific evidence indicating that this association is likely [9]. Some evidence from the WCRF/AICR report also relates higher intake of dairy products, low plasma vitamin E, and low plasma selenium concentrations with an increase in the risk of prostate cancer [9]. No consistent associations were found in the EPIC study for diets with lower nutritional quality, fat from dairy products and fruit and vegetables combined consumption. Overall, in the EPIC study, there were no associations of intake of dietary fibre, vitamin D, fish, dietary fat, alcohol, coffee, tea, and soft drinks, with the risk of prostate cancer. However, a higher risk of prostate cancer was observed with higher intake of alcohol among the O6-Methylguanine-DNA methyltransferase (MGMT) gene Ile143Val polymorphism carriers of the variant genotype in comparison with the MGMT Ile143Ile common genotype with lower alcohol intake [53]. Two mendelian randomisation studies on alcohol intake and the risk of prostate cancer yielded inconsistent results [149,150].

Results from EPIC might underestimate the role that diet may play in prostate carcinogenesis. Prostate cancer aetiology remains largely unknown and increasing evidence is being gathered supporting the relevant role of dietary factors in the disease [151,152]. According to a recent review, consumption of trans and saturated fatty acids, and food products such as processed meat contributes to prostate carcinogenesis through the alteration of prostate hormonal regulation, the induction of oxidative inflammation and stress, and the alteration of growth factor signalling and lipid metabolism. Conversely, a high consumption of fruits, vegetables, whole grain products, and fish seem to exert protective and/or therapeutic effects [151].

Biological mechanisms believed to underlie the role of diet in carcinogenesis in general include cell-cycle regulation, growth factors (such as insulin and insulin-like growth factors), immunity, inflammation, and angiogenesis [3]. Fruits and vegetables are a rich source of vitamin C and E, folate, and selenium, which are related to anti-tumorigenic effects in different cells [153]. Potential mechanisms explaining the effects of red and processed meat on cancer risk include cooking at high temperatures, which results in mutagenic potential trough the formation of DNA adducts [9]; some DNA-damaging mechanisms associated with high haem iron intake [154], and the high salt content of processed meat that may produce damage to the stomach resulting in inflammation or atrophy [9], among others. Finally, drinking alcohol can be carcinogenic to some cell types and genotoxic due to ethanol [155,156], can interfere in DNA repair mechanisms [157], and produce changes in hormone metabolism [158]. The role of diet in the aetiology of cancer will continue to be investigated in EPIC, also using tumour molecular pathology and omics research, including metabolomics, genetics, and the microbiome [3].

The EPIC study findings have contributed to the WRCF/AICR recommendations about cancer prevention. In comparison with the previous review [8], this updated article shows EPIC findings derived from longer follow-up studies that best reflect what is etiologically relevant. This review compiles novel and stronger evidence that confirms some weak associations described previously [8], such as the association of fruit and/or vegetables consumption with colorectal, breast, and prostate cancer, or dietary fibre intake with colorectal cancer. In the past years, more evidence has accumulated about dietary patterns (for example, Mediterranean diet, lower nutritional quality diet or inflammatory score of the diet), and other dietary exposures such as nutritional biomarkers (for example, several vitamins, carotenes, or fatty acids). New evidence has emerged regarding yogurt, milk, and soft drink consumption in relation to colorectal cancer risk; fatty fish and coffee intake and breast cancer risk; and yogurt and fat intakes with prostate cancer risk. Overall, this work adds ninety new published articles about colorectal, breast, lung, or prostate cancer. This reflects the remarkable increase in the available evidence from the EPIC study in the last ten years (*n* = 20 vs. *n* = 110).

The EPIC study has been highly prolific and a major source of evidence regarding the aetiology of cancer and other chronic diseases. The most relevant strengths of the EPIC study are its very large sample size, its prospective nature, the availability of blood samples to explore biomarkers, and the very comprehensive dietary questionnaires, from diverse European populations with different predominant dietary patterns and a wide variation in cancer incidence. Nevertheless, the EPIC study also has limitations that could affect its results. Like in most observational studies, our results are in partly based on self-reported data that may be subject to misclassification or measurement error in dietary intake.

## 5. Conclusions

The EPIC study has resulted in numerous high-quality publications about the role of diet in cancer prevention that help inform public health recommendations, policies, and strategies. Overall, fruit and vegetable consumption had a protective effect against colorectal, breast, and lung cancer, whereas only fruit had a protective effect against prostate cancer. A higher consumption of fish and lower consumption of red and processed meat were related with a lower risk of colorectal cancer; and higher consumption of fatty fish with lower risk of breast cancer. Calcium and yogurt intake were found to protect against colorectal and prostate cancer. Alcohol consumption increased the risk for colorectal and breast cancer. Finally, adherence to the Mediterranean diet emerged as a protective factor for colorectal and breast cancer.

## Data Availability

Not applicable.

## Supplementary Materials

The following are available online at https://www.mdpi.com/article/10.3390/nu13103582/s1, Table S1: Search terms used in databases; Table S2: Joanna Briggs Institute Critical Appraisal Tool for Systematic Reviews; Table S3: Quality assessment of included articles.

## References

[1] Sung H., Ferlay J., Siegel R.L., Laversanne M., Soerjomataram I., Jemal A., Bray F. (2021). Global Cancer Statistics 2020: GLOBOCAN Estimates of Incidence and Mortality Worldwide for 36 Cancers in 185 Countries. CA Cancer J. Clin..

[2] Randi G., Dyba T., Martos C., Giusti F., Dimitrova N., Neamtiu L., Flego M., Nicholson N., Carvalho R., Bettio M. (2020). Estimated Cancer Incidence and Mortality in Europe for the Year 2020. Eur J. Public Health.

[3] International Agency for Research on Cancer—World Health Organization (2020). World Cancer Report: Cancer Research for Cancer Prevention.

[4] Anand P., Kunnumakara A.B., Sundaram C., Harikumar K.B., Tharakan S.T., Lai O.S., Sung B., Aggarwal B.B. (2008). Cancer Is a Preventable Disease That Requires Major Lifestyle Changes. Pharm. Res..

[5] Bingham S., Riboli E. (2004). Diet and Cancer—The European Prospective Investigation into Cancer and Nutrition. Nat. Rev. Cancer.

[6] Morze J., Danielewicz A., Przybyłowicz K., Zeng H., Hoffmann G., Schwingshackl L. (2020). An Updated Systematic Review and Meta-Analysis on Adherence to Mediterranean Diet and Risk of Cancer. Eur. J. Nutr..

[7] Stein C.J., Colditz G.A. (2004). Modifiable Risk Factors for Cancer. Br. J. Cancer.

[8] Gonzalez C.A., Riboli E., Overvad K., Tjonneland A., Clavel-Chapelon F., Kaaks R., Boeing H., Trichopoulou A., Palli D., Krogh V. (2010). Diet and Cancer Prevention: Contributions from the European Prospective Investigation into Cancer and Nutrition (EPIC) Study. Eur. J. Cancer.

[9] World Cancer Research Fund/American Institute for Cancer Research (2018). Diet, Nutrition, Physical Activity and Cancer: A Global Perspective.

[10] Riboli E., Kaaks R. (1997). The EPIC Project: Rationale and Study Design. Int. J. Epidemiol..

[11] Riboli E., Hunt K.J., Slimani N., Ferrari P., Norat T., Fahey M., Charrondière U.R., Hémon B., Casagrande C., Vignat J. (2002). European Prospective Investigation into Cancer and Nutrition (EPIC): Study Populations and Data Collection. Public Health Nutr..

[12] Margetts B.M., Pietinen P. (1997). European Prospective Investigation into Cancer and Nutrition: Validity Studies on Dietary Assessment Methods. Int. J. Epidemiol..

[13] Hjartåker A., Andersen L.F., Lund E. (2007). Comparison of Diet Measures from a Food-Frequency Questionnaire with Measures from Repeated 24-Hour Dietary Recalls. The Norwegian Women and Cancer Study. Public Health Nutr..

[14] Slimani N., Kaaks R., Ferrari P., Casagrande C., Clavel-Chapelon F., Lotze G., Kroke A., Trichopoulos D., Trichopoulou A., Lauria C. (2002). European Prospective Investigation into Cancer and Nutrition (EPIC) Calibration Study: Rationale, Design and Population Characteristics. Public Health Nutr..

[15] Slimani N., Deharveng G., Unwin I., Southgate D.A.T., Vignat J., Skeie G., Salvini S., Parpinel M., Møller A., Ireland J. (2007). The EPIC Nutrient Database Project (ENDB): A First Attempt to Standardize Nutrient Databases across the 10 European Countries Participating in the EPIC Study. Eur. J. Clin. Nutr..

[16] Page M.J., McKenzie J.E., Bossuyt P.M., Boutron I., Hoffmann T.C., Mulrow C.D., Shamseer L., Tetzlaff J.M., Akl E.A., Brennan S.E. (2021). The PRISMA 2020 Statement: An Updated Guideline for Reporting Systematic Reviews. BMJ.

[17] Moola S., Munn Z., Tufanaru C., Aromataris E., Sears K., Sfetcu R., Currie M., Lisy K., Qureshi R., Mattis P., Aromataris E., Munn Z. (2020). Chapter 7: Systematic reviews of etiology and risk. JBI Manual for Evidence Synthesis.

[18] Lin K.Y., Edbrooke L., Granger C.L., Denehy L., Frawley H.C. (2019). The Impact of Gynaecological Cancer Treatment on Physical Activity Levels: A Systematic Review of Observational Studies. Braz. J. Phys. Ther..

[19] Lukomskyj N., Shi Y., Allman-Farinelli M., Rangan A. (2021). Associations between Breakfast Consumption from Childhood to Adulthood and Cardiometabolic Health: A Systematic Review. Nutr. Diet..

[20] Larkin D., Lopez V., Aromataris E. (2012). Non-pharmacological interventions for cancer-related fatigue in men treated for prostate cancer: A systematic review. JBI Libr. Syst. Rev..

[21] Van Duijnhoven F.J., Bueno-De-Mesquita H.B., Ferrari P., Jenab M., Boshuizen H.C., Ros M.M., Casagrande C., Tjønneland A., Olsen A., Overvad K. (2009). Fruit, Vegetables, and Colorectal Cancer Risk: The European Prospective Investigation into Cancer and Nutrition. Am. J. Clin. Nutr..

[22] Leenders M., Siersema P.D., Overvad K., Tjønneland A., Olsen A., Boutron-Ruault M.-C., Bastide N., Fagherazzi G., Katzke V., Kühn T. (2015). Subtypes of Fruit and Vegetables, Variety in Consumption and Risk of Colon and Rectal Cancer in the European Prospective Investigation into Cancer and Nutrition. Int. J. Cancer.

[23] Bingham S.A., Luben R., Welch A., Wareham N., Khaw K.T., Day N. (2003). Are Imprecise Methods Obscuring a Relation between Fat and Breast Cancer?. Lancet.

[24] Bingham S.A., Norat T., Moskal A., Ferrari P., Slimani N., Clavel-Chapelon F., Kesse E., Nieters A., Boeing H., Tjønneland A. (2005). Is the Association with Fiber from Foods in Colorectal Cancer Confounded by Folate Intake?. Cancer Epidemiol. Biomark. Prev..

[25] Murphy N., Norat T., Ferrari P., Jenab M., Bueno-de-Mesquita B., Skeie G., Dahm C.C., Overvad K., Olsen A., Tjønneland A. (2012). Dietary Fibre Intake and Risks of Cancers of the Colon and Rectum in the European Prospective Investigation into Cancer and Nutrition (EPIC). PLoS ONE.

[26] Jenab M., Ferrari P., Slimani N., Norat T., Casagrande C., Overad K., Olsen A., Stripp C., Tjønneland A., Boutron-Ruault M.-C. (2004). Association of Nut and Seed Intake with Colorectal Cancer Risk in the European Prospective Investigation into Cancer and Nutrition. Cancer Epidemiol. Biomark. Prev..

[27] Aglago E.K., Huybrechts I., Murphy N., Casagrande C., Nicolas G., Pischon T., Fedirko V., Severi G., Boutron-Ruault M.-C., Fournier A. (2020). Consumption of Fish and Long-Chain n-3 Polyunsaturated Fatty Acids Is Associated With Reduced Risk of Colorectal Cancer in a Large European Cohort. Clin. Gastroenterol. Hepatol..

[28] Norat T., Bingham S., Ferrari P., Slimani N., Jenab M., Mazuir M., Overvad K., Olsen A., Tjønneland A., Clavel F. (2005). Meat, Fish, and Colorectal Cancer Risk: The European Prospective Investigation into Cancer and Nutrition. J. Natl. Cancer Inst..

[29] Ward H.A., Norat T., Overvad K., Dahm C.C., Bueno-de-Mesquita H.B., Jenab M., Fedirko V., van Duijnhoven F.J.B., Skeie G., Romaguera-Bosch D. (2016). Pre-Diagnostic Meat and Fibre Intakes in Relation to Colorectal Cancer Survival in the European Prospective Investigation into Cancer and Nutrition. Br. J. Nutr..

[30] Jenab M., Bueno-de-Mesquita H.B., Ferrari P., van Duijnhoven F.J.B., Norat T., Pischon T., Jansen E.H.J.M., Slimani N., Byrnes G., Rinaldi S. (2010). Association between Pre-Diagnostic Circulating Vitamin D Concentration and Risk of Colorectal Cancer in European Populations:A Nested Case-Control Study. BMJ.

[31] Murphy N., Norat T., Ferrari P., Jenab M., Bueno-de-Mesquita B., Skeie G., Olsen A., Tjønneland A., Dahm C.C., Overvad K. (2013). Consumption of Dairy Products and Colorectal Cancer in the European Prospective Investigation into Cancer and Nutrition (EPIC). PLoS ONE.

[32] Pala V., Sieri S., Berrino F., Vineis P., Sacerdote C., Palli D., Masala G., Panico S., Mattiello A., Tumino R. (2011). Yogurt Consumption and Risk of Colorectal Cancer in the Italian European Prospective Investigation into Cancer and Nutrition Cohort. Int. J. Cancer.

[33] Sieri S., Agnoli C., Pala V., Mattiello A., Panico S., Masala G., Assedi M., Tumino R., Frasca G., Sacerdote C. (2015). Dietary habits and cancer: The experience of epicitaly. Epidemiol. Prev..

[34] Agnoli C., Grioni S., Sieri S., Palli D., Masala G., Sacerdote C., Vineis P., Tumino R., Giurdanella M.C., Pala V. (2013). Italian Mediterranean Index and Risk of Colorectal Cancer in the Italian Section of the EPIC Cohort. Int. J. Cancer.

[35] Bamia C., Lagiou P., Buckland G., Grioni S., Agnoli C., Taylor A.J., Dahm C.C., Overvad K., Olsen A., Tjønneland A. (2013). Mediterranean Diet and Colorectal Cancer Risk: Results from a European Cohort. Eur. J. Epidemiol..

[36] Deschasaux M., Huybrechts I., Julia C., Hercberg S., Egnell M., Srour B., Kesse-Guyot E., Latino-Martel P., Biessy C., Casagrande C. (2020). Association between Nutritional Profiles of Foods Underlying Nutri-Score Front-of-Pack Labels and Mortality: EPIC Cohort Study in 10 European Countries. BMJ.

[37] Jakszyn P., Cayssials V., Buckland G., Perez-Cornago A., Weiderpass E., Boeing H., Bergmann M.M., Vulcan A., Ohlsson B., Masala G. (2020). Inflammatory Potential of the Diet and Risk of Colorectal Cancer in the European Prospective Investigation into Cancer and Nutrition Study. Int. J. Cancer.

[38] Bendinelli B., Palli D., Assedi M., Facchini L., Grioni S., Agnoli C., Ricceri F., Macciotta A., Panico S., Mattiello A. (2020). Alcohol, Smoking and Rectal Cancer Risk in a Mediterranean Cohort of Adults: The European Prospective Investigation into Cancer and Nutrition (EPIC)-Italy Cohort. Eur. J. Gastroenterol. Hepatol..

[39] Murphy N., Ward H.A., Jenab M., Rothwell J.A., Boutron-Ruault M.-C., Carbonnel F., Kvaskoff M., Kaaks R., Kühn T., Boeing H. (2019). Heterogeneity of Colorectal Cancer Risk Factors by Anatomical Subsite in 10 European Countries: A Multinational Cohort Study. Clin. Gastroenterol. Hepatol..

[40] Aleksandrova K., Pischon T., Jenab M., Bueno-de-Mesquita H.B., Fedirko V., Norat T., Romaguera D., Knüppel S., Boutron-Ruault M.C., Dossus L. (2014). Combined Impact of Healthy Lifestyle Factors on Colorectal Cancer: A Large European Cohort Study. BMC Med..

[41] Eussen S.J.P.M., Vollset S.E., Igland J., Meyer K., Fredriksen A., Ueland P.M., Jenab M., Slimani N., Boffetta P., Overvad K. (2010). Plasma Folate, Related Genetic Variants, and Colorectal Cancer Risk in EPIC. Cancer Epidemiol. Biomark. Prev..

[42] Ferrari P., Jenab M., Norat T., Moskal A., Slimani N., Olsen A., Tjønneland A., Overvad K., Jensen M.K., Boutron-Ruault M.-C. (2007). Lifetime and Baseline Alcohol Intake and Risk of Colon and Rectal Cancers in the European Prospective Investigation into Cancer and Nutrition (EPIC). Int. J. Cancer.

[43] Park J.Y., Mitrou P.N., Dahm C.C., Luben R.N., Wareham N.J., Khaw K.-T., Rodwell S.A. (2009). Baseline Alcohol Consumption, Type of Alcoholic Beverage and Risk of Colorectal Cancer in the European Prospective Investigation into Cancer and Nutrition-Norfolk Study. Cancer Epidemiol..

[44] Mullee A., Romaguera D., Pearson-Stuttard J., Viallon V., Stepien M., Freisling H., Fagherazzi G., Mancini F.R., Boutron-Ruault M.-C., Kühn T. (2019). Association between Soft Drink Consumption and Mortality in 10 European Countries. JAMA Intern. Med..

[45] Gibbs D.C., Bostick R.M., McCullough M.L., Um C.Y., Flanders W.D., Jenab M., Weiderpass E., Gylling B., Gram I.T., Heath A.K. (2020). Association of Prediagnostic Vitamin D Status with Mortality among Colorectal Cancer Patients Differs by Common, Inherited Vitamin D-Binding Protein Isoforms. Int. J. Cancer.

[46] Fedirko V., Riboli E., Tjønneland A., Ferrari P., Olsen A., Bueno-de-Mesquita H.B., van Duijnhoven F.J.B.F.J.B., Norat T., Jansen E.H.J.M.E.H.J.M., Dahm C.C. (2012). Prediagnostic 25-Hydroxyvitamin D, VDR and CASR Polymorphisms, and Survival in Patients with Colorectal Cancer in Western European Populations. Cancer Epidemiol. Biomark. Prev..

[47] Eussen S.J.P.M., Vollset S.E., Hustad S., Midttun Ø., Meyer K., Fredriksen A., Ueland P.M., Jenab M., Slimani N., Boffetta P. (2010). Plasma Vitamins B2, B6, and B12, and Related Genetic Variants as Predictors of Colorectal Cancer Risk. Cancer Epidemiol. Biomark. Prev..

[48] Leenders M., Leufkens A.M., Siersema P.D., van Duijnhoven F.J.B., Vrieling A., Hulshof P.J.M., van Gils C.H., Overvad K., Roswall N., Kyrø C. (2014). Plasma and Dietary Carotenoids and Vitamins A, C and E and Risk of Colon and Rectal Cancer in the European Prospective Investigation into Cancer and Nutrition. Int. J. Cancer.

[49] Kyrø C., Olsen A., Landberg R., Skeie G., Loft S., Aman P., Leenders M., Dik V.K., Siersema P.D., Pischon T. (2014). Plasma Alkylresorcinols, Biomarkers of Whole-Grain Wheat and Rye Intake, and Incidence of Colorectal Cancer. J. Natl. Cancer Inst..

[50] Nitter M., Norgård B., de Vogel S., Eussen S.J.P.M., Meyer K., Ulvik A., Ueland P.M., Nygård O., Vollset S.E., Bjørge T. (2014). Plasma Methionine, Choline, Betaine, and Dimethylglycine in Relation to Colorectal Cancer Risk in the European Prospective Investigation into Cancer and Nutrition (EPIC). Ann. Oncol..

[51] Rothwell J.A., Murphy N., Bešević J., Kliemann N., Jenab M., Ferrari P., Achaintre D., Gicquiau A., Vozar B., Scalbert A. (2020). Metabolic Signatures of Healthy Lifestyle Patterns and Colorectal Cancer Risk in a European Cohort. Clin. Gastroenterol. Hepatol..

[52] Stepien M., Jenab M., Freisling H., Becker N.-P., Czuban M., Tjønneland A., Olsen A., Overvad K., Boutron-Ruault M.-C., Mancini F.R. (2017). Pre-Diagnostic Copper and Zinc Biomarkers and Colorectal Cancer Risk in the European Prospective Investigation into Cancer and Nutrition Cohort. Carcinogenesis.

[53] Loh Y.H., Mitrou P.N., Bowman R., Wood A., Jeffery H., Luben R., Khaw K.-T., Rodwell S. (2010). MGMT ILE143VAL Polymorphism, Dietary Factors, and the Risk of Breast, Colorectal, and Prostate Cancer in the European Prospective Investigation Into Cancer and Nutrition (EPIC)-Norfolk Study. DNA Repair.

[54] Li K., Kaaks R., Linseisen J., Rohrmann S. (2011). Dietary Calcium and Magnesium Intake in Relation to Cancer Incidence and Mortality in a German Prospective Cohort (EPIC-Heidelberg). Cancer Causes Control.

[55] Linseisen J., Grundmann N., Zoller D., Kuehn T., Jansen E.H.J.M., Chajes V., Fedirko V., Weiderpass E., Dahm C.C., Overvad K. (2021). Red Blood Cell Fatty Acids and Risk of Colorectal Cancer in the European Prospective Investigation into Cancer and Nutrition (EPIC). Cancer Epidemiol. Biomark. Prev..

[56] Kesse E., Clavel-Chapelon F., Boutron-Ruault M.C. (2006). Dietary Patterns and Risk of Colorectal Tumors: A Cohort of French Women of the National Education System (E3N). Am. J. Epidemiol..

[57] Key T.J., Appleby P.N., Spencer E.A., Travis R.C., Roddam A.W., Allen N.E., TJ K., PN A., EA S., RC T. (2009). Mortality in British Vegetarians: Results from the European Prospective Investigation into Cancer and Nutrition (EPIC-Oxford). Am. J. Clin. Nutr..

[58] Jenab M., Riboli E., Cleveland R.J., Norat T., Rinaldi S., Nieters A., Biessy C., Tjønneland A., Olsen A., Overvad K. (2007). Serum C-Peptide, IGFBP-1 and IGFBP-2 and Risk of Colon and Rectal Cancers in the European Prospective Investigation into Cancer and Nutrition. Int. J. Cancer.

[59] Ward H.A., Kuhnle G.G.C., Mulligan A.A., Lentjes M.A.H., Luben R.N., Khaw K.-T., HA W., GG K., AA M., MA L. (2010). Breast, Colorectal, and Prostate Cancer Risk in the European Prospective Investigation into Cancer and Nutrition-Norfolk in Relation to Phytoestrogen Intake Derived from an Improved Database. Am. J. Clin. Nutr..

[60] Hughes D.J., Fedirko V., Jenab M., Schomburg L., Méplan C., Freisling H., Bueno-de-Mesquita H.B., Hybsier S., Becker N.-P., Czuban M. (2015). Selenium Status Is Associated with Colorectal Cancer Risk in the European Prospective Investigation of Cancer and Nutrition Cohort. Int. J. Cancer.

[61] Vece M.M., Agnoli C., Grioni S., Sieri S., Pala V., Pellegrini N., Frasca G., Tumino R., Mattiello A., Panico S. (2015). Dietary Total Antioxidant Capacity and Colorectal Cancer in the Italian EPIC Cohort. PLoS ONE.

[62] Moskal A., Freisling H., Byrnes G., Assi N., Fahey M.T., Jenab M., Ferrari P., Tjønneland A., Petersen K.E., Dahm C.C. (2016). Main Nutrient Patterns and Colorectal Cancer Risk in the European Prospective Investigation into Cancer and Nutrition Study. Br. J. Cancer.

[63] Zamora-Ros R., Barupal D.K., Rothwell J.A., Jenab M., Fedirko V., Romieu I., Aleksandrova K., Overvad K., Kyrø C., Tjønneland A. (2017). Dietary Flavonoid Intake and Colorectal Cancer Risk in the European Prospective Investigation into Cancer and Nutrition (EPIC) Cohort. Int. J. Cancer.

[64] Zamora-Ros R., Cayssials V., Jenab M., Rothwell J.A., Fedirko V., Aleksandrova K., Tjønneland A., Kyrø C., Overvad K., Boutron-Ruault M.-C. (2018). Dietary Intake of Total Polyphenol and Polyphenol Classes and the Risk of Colorectal Cancer in the European Prospective Investigation into Cancer and Nutrition (EPIC) Cohort. Eur. J. Epidemiol..

[65] Engeset D., Dyachenko A., Ciampi A., Lund E. (2009). Dietary Patterns and Risk of Cancer of Various Sites in the Norwegian European Prospective Investigation into Cancer and Nutrition Cohort: The Norwegian Women and Cancer Study. Eur. J. Cancer Prev..

[66] Emaus M.J., Peeters P.H.M., Bakker M.F., Overvad K., Tjønneland A., Olsen A., Romieu I., Ferrari P., Dossus L., Boutron-Ruault M.C. (2016). Vegetable and Fruit Consumption and the Risk of Hormone Receptor-Defined Breast Cancer in the EPIC Cohort. Am. J. Clin. Nutr..

[67] Masala G., Assedi M., Bendinelli B., Ermini I., Sieri S., Grioni S., Sacerdote C., Ricceri F., Panico S., Mattiello A. (2012). Fruit and Vegetables Consumption and Breast Cancer Risk: The EPIC Italy Study. Breast Cancer Res. Treat..

[68] van Gils C.H., Peeters P.H.M., Bueno-de-Mesquita H.B., Boshuizen H.C., Lahmann P.H., Clavel-Chapelon F., Thiébaut A., Kesse E., Sieri S., Palli D. (2005). Consumption of Vegetables and Fruits and Risk of Breast Cancer. JAMA.

[69] Ferrari P., Rinaldi S., Jenab M., Lukanova A., Olsen A., Tjønneland A., Overvad K., Clavel-Chapelon F., Fagherazzi G., Touillaud M. (2013). Dietary Fiber Intake and Risk of Hormonal Receptor-Defined Breast Cancer in the European Prospective Investigation into Cancer and Nutrition Study. Am. J. Clin. Nutr..

[70] Engeset D., Alsaker E., Lund E., Welch A., Khaw K.-T., Clavel-Chapelon F., Thiébaut A., Chajès V., Key T.J., Allen N.E. (2006). Fish Consumption and Breast Cancer Risk. The European Prospective Investigation into Cancer and Nutrition (EPIC). Int. J. Cancer.

[71] Pala V., Krogh V., Berrino F., Sieri S., Grioni S., Tjønneland A., Olsen A., Jakobsen M.U., Overvad K., Clavel-Chapelon F. (2009). Meat, Eggs, Dairy Products, and Risk of Breast Cancer in the European Prospective Investigation into Cancer and Nutrition (EPIC) Cohort. Am. J. Clin. Nutr..

[72] Abbas S., Linseisen J., Rohrmann S., Chang-Claude J., Peeters P.H., Engel P., Brustad M., Lund E., Skeie G., Olsen A. (2013). Dietary Intake of Vitamin D and Calcium and Breast Cancer Risk in the European Prospective Investigation into Cancer and Nutrition. Nutr. Cancer.

[73] Trichopoulou A., Bamia C., Lagiou P., Trichopoulos D. (2010). Conformity to Traditional Mediterranean Diet and Breast Cancer Risk in the Greek EPIC (European Prospective Investigation into Cancer and Nutrition) Cohort. Am. J. Clin. Nutr..

[74] Buckland G., Travier N., Cottet V., González C.A., Luján-Barroso L., Agudo A., Trichopoulou A., Lagiou P., Trichopoulos D., Peeters P.H. (2013). Adherence to the Mediterranean Diet and Risk of Breast Cancer in the European Prospective Investigation into Cancer and Nutrition Cohort Study. Int. J. Cancer.

[75] Travis R.C., Allen N.E., Appleby P.N., Spencer E.A., Roddam A.W., Key T.J. (2008). A Prospective Study of Vegetarianism and Isoflavone Intake in Relation to Breast Cancer Risk in British Women. Int. J. Cancer.

[76] Heath A.K., Muller D.C., van den Brandt P.A., Papadimitriou N., Critselis E., Gunter M., Vineis P., Weiderpass E., Fagherazzi G., Boeing H. (2020). Nutrient-Wide Association Study of 92 Foods and Nutrients and Breast Cancer Risk. Breast Cancer Res..

[77] Romieu I., Scoccianti C., Chajès V., de Batlle J., Biessy C., Dossus L., Baglietto L., Clavel-Chapelon F., Overvad K., Olsen A. (2015). Alcohol Intake and Breast Cancer in the European Prospective Investigation into Cancer and Nutrition. Int. J. Cancer.

[78] Assi N., Rinaldi S., Viallon V., Dashti S.G., Dossus L., Fournier A., Cervenka I., Kvaskoff M., Turzanski-Fortner R., Bergmann M. (2020). Mediation Analysis of the Alcohol-Postmenopausal Breast Cancer Relationship by Sex Hormones in the EPIC Cohort. Int. J. Cancer.

[79] Bhoo-Pathy N., Peeters P.H.M., Uiterwaal C.S.P.M., Bueno-de-Mesquita H.B., Bulgiba A.M., Bech B.H., Overvad K., Tjønneland A., Olsen A., Clavel-Chapelon F. (2015). Coffee and Tea Consumption and Risk of Pre- and Postmenopausal Breast Cancer in the European Prospective Investigation into Cancer and Nutrition (EPIC) Cohort Study. Breast Cancer Res..

[80] Assi N., Moskal A., Slimani N., Viallon V., Chajes V., Freisling H., Monni S., Knueppel S., Förster J., Weiderpass E. (2016). A Treelet Transform Analysis to Relate Nutrient Patterns to the Risk of Hormonal Receptor-Defined Breast Cancer in the European Prospective Investigation into Cancer and Nutrition (EPIC). Public Health Nutr..

[81] Bakker M.F., Peeters P.H., Klaasen V.M., Bueno-de-Mesquita H.B., Jansen E.H., Ros M.M., Travier N., Olsen A., Tjønneland A., Overvad K. (2016). Plasma Carotenoids, Vitamin C, Tocopherols, and Retinol and the Risk of Breast Cancer in the European Prospective Investigation into Cancer and Nutrition Cohort. Am. J. Clin. Nutr..

[82] Matta M., Huybrechts I., Biessy C., Casagrande C., Yammine S., Fournier A., Olsen K.S., Lukic M., Gram I.T., Ardanaz E. (2021). Dietary Intake of Trans Fatty Acids and Breast Cancer Risk in 9 European Countries. BMC Med..

[83] Chajès V., Thiébaut A.C.M., Rotival M., Gauthier E., Maillard V., Boutron-Ruault M.-C., Joulin V., Lenoir G.M., Clavel-Chapelon F.F., Chajes V. (2008). Association between Serum Trans-Monounsaturated Fatty Acids and Breast Cancer Risk in the E3N-EPIC Study. Am. J. Epidemiol..

[84] Chajès V., Assi N., Biessy C., Ferrari P., Rinaldi S., Slimani N., Lenoir G.M., Baglietto L., His M., Boutron-Ruault M.C. (2017). A Prospective Evaluation of Plasma Phospholipid Fatty Acids and Breast Cancer Risk in the EPIC Study. Ann. Oncol..

[85] Romieu I., Ferrari P., Rinaldi S., Slimani N., Jenab M., Olsen A., Tjonneland A., Overvad K., Boutron-Ruault M.-C., Lajous M. (2012). Dietary Glycemic Index and Glycemic Load and Breast Cancer Risk in the European Prospective Investigation into Cancer and Nutrition (EPIC). Am. J. Clin. Nutr..

[86] Sieri S., Pala V., Brighenti F., Agnoli C., Grioni S., Berrino F., Scazzina F., Palli D., Masala G., Vineis P. (2013). High Glycemic Diet and Breast Cancer Occurrence in the Italian EPIC Cohort. Nutr. Metab. Cardiovasc. Dis..

[87] Spencer E.A., Key T.J., Appleby P.N., van Gils C.H., Olsen A., Tjønneland A., Clavel-Chapelon F., Boutron-Ruault M.-C., Touillaud M., Sánchez M.-J. (2009). Prospective Study of the Association between Grapefruit Intake and Risk of Breast Cancer in the European Prospective Investigation into Cancer and Nutrition (EPIC). Cancer Causes Control.

[88] Buckland G., Travier N., Agudo A., Fonseca-Nunes A., Navarro C., Lagiou P., Demetriou C., Amiano P., Dorronsoro M., Chirlaque M.-D. (2012). Olive Oil Intake and Breast Cancer Risk in the Mediterranean Countries of the European Prospective Investigation into Cancer and Nutrition Study. Int. J. Cancer.

[89] Tjønneland A., Christensen J., Olsen A., Stripp C., Thomsen B.L., Overvad K., Peeters P.H.M., van Gils C.H., Bueno-de-Mesquita H.B., Ocké M.C. (2007). Alcohol Intake and Breast Cancer Risk: The European Prospective Investigation into Cancer and Nutrition (EPIC). Cancer Causes Control.

[90] Masala G., Bendinelli B., Assedi M., Occhini D., Zanna I., Sieri S., Agnoli C., Sacerdote C., Ricceri F., Mattiello A. (2017). Up to One-Third of Breast Cancer Cases in Post-Menopausal Mediterranean Women Might Be Avoided by Modifying Lifestyle Habits: The EPIC Italy Study. Breast Cancer Res. Treat..

[91] Romieu I., Ferrari P., Chajès V., de Batlle J., Biessy C., Scoccianti C., Dossus L., Christine Boutron M., Bastide N., Overvad K. (2017). Fiber Intake Modulates the Association of Alcohol Intake with Breast Cancer. Int. J. Cancer.

[92] Thiebaut A.C.M., Clavel-Chapelon F. (2001). Fat Consumption and Breast Cancer: Preliminary Results from the E3N-Epic Cohort. Bull. Cancer.

[93] Keinan-Boker L., van Der Schouw Y.T., Grobbee D.E., Peeters P.H.M. (2004). Dietary Phytoestrogens and Breast Cancer Risk. Am. J. Clin. Nutr..

[94] Sieri S., Krogh V., Ferrari P., Berrino F., Pala V., Thiébaut A.C.M., Tjønneland A., Olsen A., Overvad K., Jakobsen M.U. (2008). Dietary Fat and Breast Cancer Risk in the European Prospective Investigation into Cancer and Nutrition. Am. J. Clin. Nutr..

[95] Maillard V., Kuriki K., Lefebvre B., Boutron-Ruault M.-C., Lenoir G.M., Joulin V., Clavel-Chapelon F., Chajès V. (2010). Serum Carotenoid, Tocopherol and Retinol Concentrations and Breast Cancer Risk in the E3N-EPIC Study. Int. J. Cancer.

[96] Nagel G., Linseisen J., van Gils C.H., Peeters P.H., Boutron-Ruault M.C., Clavel-Chapelon F., Romieu I., Tjønneland A., Olsen A., Roswall N. (2010). Dietary Beta-Carotene, Vitamin C and E Intake and Breast Cancer Risk in the European Prospective Investigation into Cancer and Nutrition (EPIC). Breast Cancer Res. Treat..

[97] Zamora-Ros R., Ferrari P., González C.A., Tjønneland A., Olsen A., Bredsdorff L., Overvad K., Touillaud M., Perquier F., Fagherazzi G. (2013). Dietary Flavonoid and Lignan Intake and Breast Cancer Risk According to Menopause and Hormone Receptor Status in the European Prospective Investigation into Cancer and Nutrition (EPIC) Study. Breast Cancer Res. Treat..

[98] De Batlle J., Ferrari P., Chajes V., Park J.Y., Slimani N., McKenzie F., Overvad K., Roswall N., Tjønneland A., Boutron-Ruault M.C. (2015). Dietary Folate Intake and Breast Cancer Risk: European Prospective Investigation into Cancer and Nutrition. J. Natl. Cancer Inst..

[99] Kyrø C., Zamora-Ros R., Scalbert A., Tjønneland A., Dossus L., Johansen C., Bidstrup P.E., Weiderpass E., Christensen J., Ward H. (2015). Pre-Diagnostic Polyphenol Intake and Breast Cancer Survival: The European Prospective Investigation into Cancer and Nutrition (EPIC) Cohort. Breast Cancer Res. Treat..

[100] Matejcic M., de Batlle J., Ricci C., Biessy C., Perrier F., Huybrechts I., Weiderpass E., Boutron-Ruault M.C., Cadeau C., His M. (2017). Biomarkers of Folate and Vitamin B12 and Breast Cancer Risk: Report from the EPIC Cohort. Int. J. Cancer.

[101] Büchner F.L., Bueno-de-Mesquita H.B., Linseisen J., Boshuizen H.C., Kiemeney L.A.L.M., Ros M.M., Overvad K., Hansen L., Tjonneland A., Raaschou-Nielsen O. (2010). Fruits and Vegetables Consumption and the Risk of Histological Subtypes of Lung Cancer in the European Prospective Investigation into Cancer and Nutrition (EPIC). Cancer Causes Control.

[102] Linseisen J., Rohrmann S., Miller A.B., Bueno-de-Mesquita H.B., Büchner F.L., Vineis P., Agudo A., Gram I.T., Janson L., Krogh V. (2007). Fruit and Vegetable Consumption and Lung Cancer Risk: Updated Information from the European Prospective Investigation into Cancer and Nutrition (EPIC). Int. J. Cancer.

[103] Miller A.B., Altenburg H.-P., Bueno-de-Mesquita B., Boshuizen H.C., Agudo A., Berrino F., Gram I.T., Janson L., Linseisen J., Overvad K. (2004). Fruits and Vegetables and Lung Cancer: Findings from the European Prospective Investigation into Cancer and Nutrition. Int. J. Cancer.

[104] Linseisen J., Rohrmann S., Bueno-de-Mesquita B., Büchner F.L., Boshuizen H.C., Agudo A., Gram I.T., Dahm C.C., Overvad K., Egeberg R. (2011). Consumption of Meat and Fish and Risk of Lung Cancer: Results from the European Prospective Investigation into Cancer and Nutrition. Cancer Causes Control.

[105] Rohrmann S., Linseisen J., Boshuizen H.C., Whittaker J., Agudo A., Vineis P., Boffetta P., Jensen M.K., Olsen A., Overvad K. (2006). Ethanol Intake and Risk of Lung Cancer in the European Prospective Investigation into Cancer and Nutrition (EPIC). Am. J. Epidemiol..

[106] Johansson M., Relton C., Ueland P.M., Vollset S.E., Midttun Ø., Nygård O., Slimani N., Boffetta P., Jenab M., Clavel-Chapelon F. (2010). Serum B Vitamin Levels and Risk of Lung Cancer. JAMA.

[107] Myint P.K., Wilson A.M., Clark A.B., Luben R.N., Wareham N.J., Khaw K.-T. (2019). Plasma Vitamin C Concentrations and Risk of Incident Respiratory Diseases and Mortality in the European Prospective Investigation into Cancer-Norfolk Population-Based Cohort Study. Eur. J. Clin. Nutr..

[108] Nimptsch K., Rohrmann S., Kaaks R., Linseisen J. (2010). Dietary Vitamin K Intake in Relation to Cancer Incidence and Mortality: Results from the Heidelberg Cohort of the European Prospective Investigation into Cancer and Nutrition (EPIC-Heidelberg). Am. J. Clin. Nutr..

[109] Ward H.A., Whitman J., Muller D.C., Johansson M., Jakszyn P., Weiderpass E., Palli D., Fanidi A., Vermeulen R., Tjønneland A. (2019). Haem Iron Intake and Risk of Lung Cancer in the European Prospective Investigation into Cancer and Nutrition (EPIC) Cohort. Eur. J. Clin. Nutr..

[110] Zuo H., Ueland P.M., Midttun Ø., Vollset S.E., Tell G.S., Theofylaktopoulou D., Travis R.C., Boutron-Ruault M.-C., Fournier A., Severi G. (2018). Results from the European Prospective Investigation into Cancer and Nutrition Link Vitamin B6 Catabolism and Lung Cancer Risk. Cancer Res..

[111] Key T.J., Allen N., Appleby P., Overvad K., Tjønneland A., Miller A., Boeing H., Karalis D., Psaltopoulou T., Berrino F. (2004). Fruits and Vegetables and Prostate Cancer: No Association among 1104 Cases in a Prospective Study of 130544 Men in the European Prospective Investigation into Cancer and Nutrition (EPIC). Int. J. Cancer.

[112] Perez-Cornago A., Travis R.C., Appleby P.N., Tsilidis K.K., Tjønneland A., Olsen A., Overvad K., Katzke V., Kühn T., Trichopoulou A. (2017). Fruit and Vegetable Intake and Prostate Cancer Risk in the European Prospective Investigation into Cancer and Nutrition (EPIC). Int. J. Cancer.

[113] Suzuki R., Allen N.E., Key T.J., Appleby P.N., Tjønneland A., Johnsen N.F., Jensen M.K., Overvad K., Boeing H., Pischon T. (2009). A Prospective Analysis of the Association between Dietary Fiber Intake and Prostate Cancer Risk in EPIC. Int. J. Cancer.

[114] Crowe F.L., Key T.J., Appleby P.N., Travis R.C., Overvad K., Jakobsen M.U., Johnsen N.F., Tjønneland A., Linseisen J., Rohrmann S. (2008). Dietary Fat Intake and Risk of Prostate Cancer in the European Prospective Investigation into Cancer and Nutrition. Am. J. Clin. Nutr..

[115] Allen N.E., Key T.J., Appleby P.N., Travis R.C., Roddam A.W., Tjønneland A., Johnsen N.F., Overvad K., Linseisen J., Rohrmann S. (2008). Animal Foods, Protein, Calcium and Prostate Cancer Risk: The European Prospective Investigation into Cancer and Nutrition. Br. J. Cancer.

[116] Rohrmann S., Linseisen J., Key T.J., Jensen M.K., Overvad K., Johnsen N.F., Tjønneland A., Kaaks R., Bergmann M.M., Weikert C. (2008). Alcohol Consumption and the Risk for Prostate Cancer in the European Prospective Investigation into Cancer and Nutrition. Cancer Epidemiol. Biomark. Prev..

[117] Sen A., Papadimitriou N., Lagiou P., Perez-Cornago A., Travis R.C., Key T.J., Murphy N., Gunter M., Freisling H., Tzoulaki I. (2019). Coffee and Tea Consumption and Risk of Prostate Cancer in the European Prospective Investigation into Cancer and Nutrition. Int. J. Cancer.

[118] Nimptsch K., Rohrmann S., Linseisen J. (2008). Dietary Intake of Vitamin K and Risk of Prostate Cancer in the Heidelberg Cohort of the European Prospective Investigation into Cancer and Nutrition (EPIC-Heidelberg). Am. J. Clin. Nutr..

[119] Nimptsch K., Rohrmann S., Nieters A., Linseisen J. (2009). Serum Undercarboxylated Osteocalcin as Biomarker of Vitamin K Intake and Risk of Prostate Cancer: A Nested Case-Control Study in the Heidelberg Cohort of the European Prospective Investigation into Cancer and Nutrition. Cancer Epidemiol. Biomark. Prev..

[120] Perez-Cornago A., Huybrechts I., Appleby P.N., Schmidt J.A., Crowe F.L., Overvad K., Tjønneland A., Kühn T., Katzke V., Trichopoulou A. (2020). Intake of Individual Fatty Acids and Risk of Prostate Cancer in the European Prospective Investigation into Cancer and Nutrition. Int. J. Cancer.

[121] Price A.J., Allen N.E., Appleby P.N., Crowe F.L., Jenab M., Rinaldi S., Slimani N., Kaaks R., Rohrmann S., Boeing H. (2010). Plasma Phytanic Acid Concentration and Risk of Prostate Cancer: Results from the European Prospective Investigation into Cancer and Nutrition. Am. J. Clin. Nutr..

[122] Allen N.E., Key T.J., Appleby P.N., Travis R.C., Roddam A.W., Rinaldi S., Egevad L., Rohrmann S., Linseisen J., Pischon T. (2007). Serum Insulin-like Growth Factor (IGF)-I and IGF-Binding Protein-3 Concentrations and Prostate Cancer Risk: Results from the European Prospective Investigation into Cancer and Nutrition. Cancer Epidemiol. Biomark. Prev..

[123] Key T.J., Appleby P.N., Allen N.E., Travis R.C., Roddam A.W., Jenab M., Egevad L., Tjønneland A., Johnsen N.F., Overvad K. (2007). Plasma Carotenoids, Retinol, and Tocopherols and the Risk of Prostate Cancer in the European Prospective Investigation into Cancer and Nutrition Study. Am. J. Clin. Nutr..

[124] Allen N.E., Appleby P.N., Roddam A.W., Tjønneland A., Johnsen N.F., Overvad K., Boeing H., Weikert S., Kaaks R., Linseisen J. (2008). Plasma Selenium Concentration and Prostate Cancer Risk: Results from the European Prospective Investigation into Cancer and Nutrition (EPIC). Am. J. Clin. Nutr..

[125] Crowe F.L., Allen N.E., Appleby P.N., Overvad K., Aardestrup I.V., Johnsen N.F., Tjønneland A., Linseisen J., Kaaks R., Boeing H. (2008). Fatty Acid Composition of Plasma Phospholipids and Risk of Prostate Cancer in a Case-Control Analysis Nested within the European Prospective Investigation into Cancer and Nutrition. Am. J. Clin. Nutr..

[126] Travis R., Crowe F., Allen N., Appleby P., Roddam A., Tjønneland A., Olsen A., Linseisen J., Kaaks R., Boeing H. (2009). Serum Vitamin D and Risk of Prostate Cancer in a Case-Control Analysis Nested within the European Prospective Investigation into Cancer and Nutrition (EPIC). Am. J. Epidemiol..

[127] Travis R.C., Spencer E.A., Allen N.E., Appleby P.N., Roddam A.W., Overvad K., Johnsen N.F., Olsen A., Kaaks R., Linseisen J. (2009). Plasma Phyto-Oestrogens and Prostate Cancer in the European Prospective Investigation into Cancer and Nutrition. Br. J. Cancer.

[128] Steinbrecher A., Rohrmann S., Timofeeva M., Risch A., Jansen E., Linseisen J. (2010). Dietary Glucosinolate Intake, Polymorphisms in Selected Biotransformation Enzymes, and Risk of Prostate Cancer. Cancer Epidemiol. Biomark. Prev..

[129] Dahm C.C., Gorst-Rasmussen A., Crowe F.L., Roswall N., Tjønneland A., Drogan D., Boeing H., Teucher B., Kaaks R., Adarakis G. (2012). Fatty Acid Patterns and Risk of Prostate Cancer in a Case-Control Study Nested within the European Prospective Investigation into Cancer and Nutrition. Am. J. Clin. Nutr..

[130] Jakszyn P.G., Allen N.E., Lujan-Barroso L., Gonzalez C.A., Key T.J., Fonseca-Nunes A., Tjønneland A., Føns-Johnsen N., Overvad K., Teucher B. (2012). Nitrosamines and Heme Iron and Risk of Prostate Cancer in the European Prospective Investigation into Cancer and Nutrition. Cancer Epidemiol. Biomark. Prev..

[131] Papadimitriou N., Muller D., van den Brandt P.A., Geybels M., Patel C.J., Gunter M.J., Lopez D.S., Key T.J., Perez-Cornago A., Ferrari P. (2020). A Nutrient-Wide Association Study for Risk of Prostate Cancer in the European Prospective Investigation into Cancer and Nutrition and the Netherlands Cohort Study. Eur. J. Nutr..

[132] Barzi A., Lenz A., Labonte M., Lenz H. (2013). Molecular Pathways: Estrogen Pathway in Colorectal Cancer. Clin. Cancer Res..

[133] Potter J., Slattery M., Bostick R., Gapstur S. (1993). Colon Cancer: A Review of the Epidemiology. Epidemiol. Rev..

[134] Dimitrakopoulou V.I., Tsilidis K.K., Haycock P.C., Dimou N.L., Al-Dabhani K., Martin R.M., Lewis S.J., Gunter M.J., Mondul A., Shui I.M. (2017). Circulating Vitamin D Concentration and Risk of Seven Cancers: Mendelian Randomisation Study. BMJ.

[135] Bouvard V., Loomis D., Guyton K.Z., Grosse Y., Ghissassi F.E., Benbrahim-Tallaa L., Guha N., Mattock H., Straif K. (2015). Carcinogenicity of Consumption of Red and Processed Meat. Lancet Oncol..

[136] Veettil S.K., Wong T.Y., Loo Y.S., Playdon M.C., Lai N.M., Giovannucci E.L., Chaiyakunapruk N. (2021). Role of Diet in Colorectal Cancer Incidence: Umbrella Review of Meta-Analyses of Prospective Observational Studies. JAMA Netw. Open.

[137] McDonald J.A., Goyal A., Terry M.B. (2013). Alcohol Intake and Breast Cancer Risk: Weighing the Overall Evidence. Curr. Breast Cancer Rep..

[138] Holmes M.D., Willett W.C. (2004). Does Diet Affect Breast Cancer Risk?. Breast Cancer Res..

[139] Zeinomar N., Knight J.A., Genkinger J.M., Phillips K.A., Daly M.B., Milne R.L., Dite G.S., Kehm R.D., Liao Y., Southey M.C. (2019). Alcohol Consumption, Cigarette Smoking, and Familial Breast Cancer Risk: Findings from the Prospective Family Study Cohort (ProF-SC). Breast Cancer Res..

[140] Hamajima N., Hirose K., Tajima K., Rohan T., Calle E.E., Heath C.W., Coates R.J., Liff J.M., Talamini R., Chantarakul N. (2002). Alcohol, Tobacco and Breast Cancer—Collaborative Reanalysis of Individual Data from 53 Epidemiological Studies, Including 58 515 Women with Breast Cancer and 95 067 Women without the Disease. Br. J. Cancer.

[141] Dandamudi A., Tommie J., Nommsen-Rivers L., Couch S. (2018). Dietary Patterns and Breast Cancer Risk: A Systematic Review. Anticancer. Res..

[142] Boyd N.F., Stone J., Vogt K.N., Connelly B.S., Martin L.J., Minkin S. (2003). Dietary Fat and Breast Cancer Risk Revisited: A Meta-Analysis of the Published Literature. Br. J. Cancer.

[143] Xia H., Ma S., Wang S., Sun G. (2015). Meta-Analysis of Saturated Fatty Acid Intake and Breast Cancer Risk. Medicine.

[144] Cao Y., Hou L., Wang W. (2016). Dietary Total Fat and Fatty Acids Intake, Serum Fatty Acids and Risk of Breast Cancer: A Meta-Analysis of Prospective Cohort Studies. Int. J. Cancer.

[145] Boeke C.E., Eliassen A.H., Chen W.Y., Cho E., Holmes M.D., Rosner B., Willett W.C., Tamimi R.M. (2014). Dietary Fat Intake in Relation to Lethal Breast Cancer in Two Large Prospective Cohort Studies. Breast Cancer Res. Treat..

[146] Luo J., Shen L., Zheng D. (2014). Association between Vitamin C Intake and Lung Cancer: A Dose-Response Meta-Analysis. Sci. Rep..

[147] Wang M., Qin S., Zhang T., Song X., Zhang S. (2015). The Effect of Fruit and Vegetable Intake on the Development of Lung Cancer: A Meta-Analysis of 32 Publications and 20 414 Cases. Eur. J. Clin. Nutr..

[148] Vieira A.R., Abar L., Vingeliene S., Chan D.S.M., Aune D., Navarro-Rosenblatt D., Stevens C., Greenwood D., Norat T. (2016). Fruits, Vegetables and Lung Cancer Risk: A Systematic Review and Meta-Analysis. Ann. Oncol..

[149] Zuccolo L., Lewis S., Hamdy F., Neal D., Donovan J., Smith G.D. (2011). O6-2.5 Alcohol and Prostate Cancer Risk: A Mendelian Randomisation Approach. J. Epidemiol. Commun. Health.

[150] Brunner C., Davies N.M., Martin R.M., Eeles R., Easton D., Kote-Jarai Z., Al Olama A.A., Benlloch S., Muir K., Giles G. (2017). Alcohol Consumption and Prostate Cancer Incidence and Progression: A Mendelian Randomisation Study. Int. J. Cancer.

[151] Oczkowski M., Dziendzikowska K., Pasternak-Winiarska A., Włodarek D., Gromadzka-Ostrowska J. (2021). Dietary Factors and Prostate Cancer Development, Progression, and Reduction. Nutrients.

[152] Wolk A. (2005). Diet, Lifestyle and Risk of Prostate Cancer. Acta Oncol..

[153] Lippmann D., Lehmann C., Florian S., Barknowitz G., Haack M., Mewis I., Wiesner M., Schreiner M., Glatt H., Brigelius-Flohé R. (2014). Glucosinolates from Pak Choi and Broccoli Induce Enzymes and Inhibit Inflammation and Colon Cancer Differently. Food Funct..

[154] Gilsing A., Fransen F., de Kok T., Goldbohm A., Schouten L., de Bruïne A., van Engeland M., van den Brandt P., de Goeij A., Weijenberg M. (2013). Dietary Heme Iron and the Risk of Colorectal Cancer with Specific Mutations in KRAS and APC. Carcinogenesis.

[155] Seitz H.K., Stickel F. (2007). Molecular Mechanisms of Alcohol-Mediated Carcinogenesis. Nat. Rev. Cancer.

[156] Albano E. (2006). Alcohol, Oxidative Stress and Free Radical Damage. Proc. Nutr. Soc..

[157] Boffetta P., Hashibe M. (2006). Alcohol and Cancer. Lancet Oncol..

[158] Hankinson S., Willett W., Manson J., Hunter D., Colditz G., Stampfer M., Longcope C., Speizer F. (1995). Alcohol, Height, and Adiposity in Relation to Estrogen and Prolactin Levels in Postmenopausal Women. J. Natl. Cancer Inst..

