# Supplementary material for: Evidence Update on the Relationship between Diet and the Most Common Cancers from the European Prospective Investigation into Cancer and Nutrition (EPIC) Study: A Systematic Review
Source: Nutrients. 2021 Oct 13;13(10):3582. doi: 10.3390/nu13103582 (PMC8540388; doi:10.3390/nu13103582)
Supplement: Supplementary file 1 [file nutrients-13-03582-s001.zip › nutrients-1393714-supplementary.pdf]

Supplementary Material

Table S1. Search terms used in databases.

**MEDLINE (via PubMed)**
((("european prospective investigation into cancer" OR "european prospective investigation into cancer and nutrition" OR "EPIC study") AND ("lung cancer" OR "prostate cancer" OR "breast cancer" OR "colorectal cancer" OR "colon cancer" OR "rectal cancer") AND ("diet" OR "intake" OR "nutrients")))
**Scopus**
(TITLE-ABS-KEY (("european prospective investigation into cancer" OR "european prospective investigation into cancer and nutrition" OR "EPIC study")) AND TITLE-ABS-KEY (("lung cancer" OR "prostate cancer" OR "breast cancer" OR "colorectal cancer" OR "colon cancer" OR "rectal cancer")) AND TITLE-ABS-KEY (("diet" OR "intake" OR "nutrients")))
**Web of Science**
(("european prospective investigation into cancer" OR "european prospective investigation into cancer and nutrition" OR "EPIC study")) AND TEMA: (("lung cancer" OR "prostate cancer" OR "breast cancer" OR "colorectal cancer" OR "colon cancer" OR "rectal cancer")) AND TEMA: (("diet" OR "intake" OR "nutrients"))

Table S2. Joanna Briggs Institute Critical Appraisal Tool for Systematic Reviews.

	Yes	No	Unclear	Not applicable
1. Were the two groups similar and recruited from the same population?	□	□	□	□
2. Were the exposures measured similarly to assign people to both exposed and unexposed groups?	□	□	□	□
3. Was the exposure measured in a valid and reliable way?	□	□	□	□
4. Were confounding factors identified?	□	□	□	□
5. Were strategies to deal with confounding factors stated?	□	□	□	□
6. Were the groups/participants free of the outcome at the start of the study (or at the moment of exposure)?	□	□	□	□
7. Were the outcomes measured in a valid and reliable way?	□	□	□	□
8. Was the follow up time reported and sufficient to be long enough for outcomes to occur?	□	□	□	□
9. Was follow up complete, and if not, were the reasons to loss to follow up described and explored?	□	□	□	□
10. Were strategies to address incomplete follow up utilized?	□	□	□	□
11. Was appropriate statistical analysis used?	□	□	□	□

Table S3. Quality assessment of included articles.

Study	Item 1	Item 2	Item 3	Item 4	Item 5	Item 6	Item 7	Item 8	Item 9	Item 10	Item 11	Quality Category
Abbas 2013 [72]	N/A	N/A	✓	✓	✓	✓	✓	✓	✓	N/A	✓	High
Allen 2007 [122]	✓	✓	✓	✓	✓	✓	✓	✓	✓	N/A	✓	High
Allen, Key 2008 [115]	×	✓	✓	✓	✓	✓	✓	✓	✓	N/A	✓	High
Allen, Naomi 2008 [124]	×	✓	✓	✓	✓	✓	✓	✓	✓	N/A	✓	High
Aglago 2020 [27]	×	✓	✓	✓	✓	✓	✓	✓	✓	N/A	✓	High
Agnoli 2013 [34]	N/A	N/A	✓	✓	✓	✓	✓	✓	✓	N/A	✓	High
Assi 2016 [80]	N/A	N/A	✓	✓	✓	✓	✓	✓	✓	N/A	✓	High
Assi 2020 [78]	×	✓	✓	✓	✓	✓	✓	✓	✓	N/A	✓	High
Aleksandrova 2014 [40]	N/A	N/A	✓	✓	✓	✓	✓	✓	✓	N/A	✓	High
Bakker 2016 [81]	✓	✓	✓	✓	✓	✓	✓	✓	✓	N/A	✓	High
Bamia 2013 [35]	N/A	N/A	✓	✓	✓	✓	✓	✓	✓	N/A	✓	High
Bendinelli 2020 [38]	×	✓	✓	✓	✓	✓	✓	✓	✓	N/A	✓	High
Bhoo-Pathy 2015 [79]	N/A	N/A	✓	✓	✓	✓	✓	✓	✓	N/A	✓	High
Bingham 2005 [24]	N/A	N/A	✓	✓	✓	✓	✓	✓	✓	N/A	✓	High
Büchner 2010 [101]	N/A	N/A	✓	✓	✓	✓	✓	✓	✓	N/A	✓	High
Buckland 2012 [88]	N/A	N/A	✓	✓	✓	✓	✓	✓	✓	N/A	✓	High
Buckland 2013 [74]	N/A	N/A	✓	✓	✓	✓	✓	✓	✓	N/A	✓	High
Chajès 2008 [83]	×	✓	✓	✓	✓	✓	✓	✓	✓	N/A	✓	High
Chajès 2017 [84]	×	✓	✓	✓	✓	✓	✓	✓	✓	N/A	✓	High
Crowe, Allen 2008 [125]	×	✓	✓	✓	✓	✓	✓	✓	✓	N/A	✓	High
Crowe, Key 2008 [114]	N/A	N/A	✓	✓	✓	✓	✓	✓	✓	N/A	✓	High
de Batlle 2015 [98]	N/A	N/A	✓	✓	✓	✓	✓	✓	✓	N/A	✓	High
Dahm 2012 [129]	N/A	N/A	✓	✓	✓	✓	✓	?	✓	N/A	✓	High
Deschasaux 2018 [36]	N/A	N/A	✓	✓	✓	✓	✓	✓	✓	N/A	✓	High
Emaus 2016 [66]	N/A	N/A	✓	✓	✓	✓	✓	✓	✓	N/A	✓	High
Engeset 2006 [70]	N/A	N/A	✓	✓	✓	✓	✓	✓	✓	N/A	✓	High
Engeset 2009 [65]	N/A	N/A	✓	✓	✓	✓	✓	✓	✓	N/A	✓	High
Eussen, Vollset, Hustad 2010 [47]	✓	✓	✓	✓	✓	✓	✓	✓	✓	N/A	✓	High
Eussen, Vollset, Igland 2010 [41]	✓	✓	✓	✓	✓	✓	✓	✓	✓	N/A	✓	High
Fedirko 2012 [46]	N/A	N/A	✓	✓	✓	✓	✓	✓	✓	N/A	✓	High
Ferrari 2007 [42]	N/A	N/A	✓	✓	✓	✓	✓	✓	✓	N/A	✓	High
Ferrari 2013 [69]	N/A	N/A	✓	✓	✓	✓	✓	✓	✓	N/A	✓	High
Gibbs 2020 [45]	×	✓	✓	✓	✓	✓	✓	✓	✓	N/A	✓	High
Heath 2020 [76]	✓	✓	✓	✓	✓	✓	✓	✓	✓	N/A	✓	High
Hughes 2015 [60]	✓	✓	✓	✓	✓	✓	✓	✓	✓	N/A	✓	High
Jakszyn 2012 [130]	N/A	N/A	✓	✓	✓	✓	✓	✓	✓	N/A	✓	High
Jakszyn 2020 [130]	N/A	N/A	✓	✓	✓	✓	✓	✓	✓	N/A	✓	High
Jenab 2004 [26]	N/A	N/A	✓	✓	✓	✓	✓	✓	✓	N/A	✓	High
Jenab 2007 [58]	✓	✓	✓	✓	✓	✓	✓	✓	✓	N/A	✓	High
Jenab 2010 [30]	✓	✓	✓	✓	✓	✓	✓	✓	✓	N/A	✓	High
Johansson 2010 [106]	?	✓	✓	✓	✓	✓	✓	?	✓	N/A	✓	High
Keinan-Boker 2004 [93]	N/A	N/A	✓	✓	✓	✓	✓	✓	✓	N/A	✓	High
Kesse 2006 [56]	N/A	N/A	✓	✓	✓	✓	✓	✓	✓	N/A	✓	High
Key 2004 [111]	N/A	N/A	✓	✓	✓	✓	✓	✓	✓	N/A	✓	High
Key 2007 [123]	✓	✓	✓	✓	✓	✓	✓	✓	✓	N/A	✓	High
Key 2009 [57]	N/A	N/A	✓	✓	✓	✓	✓	?	✓	N/A	✓	High
Kyrø 2014 [49]	✓	✓	✓	✓	✓	✓	✓	✓	✓	N/A	✓	High
Kyrø 2015 [99]	N/A	N/A	✓	✓	✓	✓	✓	✓	✓	N/A	✓	High
Leenders 2014 [48]	✓	✓	✓	✓	✓	✓	✓	✓	✓	N/A	✓	High
Leenders 2015 [22]	N/A	N/A	✓	✓	✓	✓	✓	✓	✓	N/A	✓	High
Li 2011 [54]	N/A	N/A	✓	✓	✓	✓	✓	✓	✓	N/A	✓	High
Linseisen 2007 [102]	N/A	N/A	✓	✓	✓	✓	✓	✓	✓	N/A	✓	High
Linseisen 2011 [104]	N/A	N/A	✓	✓	✓	✓	✓	✓	✓	N/A	✓	High
Linseisen 2021 [55]	×	✓	✓	✓	✓	✓	✓	✓	✓	N/A	✓	High
Loh 2010 [53]	×	✓	✓	✓	✓	✓	✓	?	✓	N/A	✓	High
Maillard 2010 [95]	✓	✓	✓	✓	✓	✓	✓	✓	✓	N/A	✓	High
Masala 2012 [67]	N/A	N/A	✓	✓	✓	✓	✓	✓	✓	N/A	✓	High
Masala 2017 [90]	✓	✓	✓	✓	✓	✓	✓	✓	✓	N/A	✓	High
Matejcic 2017 [100]	×	✓	✓	✓	✓	✓	✓	✓	✓	N/A	✓	High
Matta 2021 [82]	N/A	N/A	✓	✓	✓	✓	✓	✓	✓	N/A	✓	High
Miller 2004 [103]	N/A	N/A	✓	✓	✓	✓	✓	?	✓	N/A	✓	High

Moskal 2016 [62]	N/A	N/A	✓	✓	✓	✓	✓	✓	✓	N/A	✓	High
Mullee 2019 [44]	N/A	N/A	✓	✓	✓	✓	✓	✓	✓	N/A	✓	High
Murphy 2012 [25]	N/A	N/A	✓	✓	✓	✓	✓	✓	✓	N/A	✓	High
Murphy 2013 [31]	N/A	N/A	✓	✓	✓	✓	✓	✓	✓	N/A	✓	High
Murphy 2019 [39]	N/A	N/A	✓	✓	✓	✓	✓	✓	✓	N/A	✓	High
Myint 2019 [107]	N/A	N/A	✓	✓	✓	✓	✓	✓	✓	N/A	✓	High
Nagel 2010 [96]	N/A	N/A	✓	✓	✓	✓	✓	✓	✓	N/A	✓	High
Nimptsch 2008 [118]	N/A	N/A	✓	✓	✓	✓	✓	✓	✓	N/A	✓	High
Nimptsch 2009 [119]	✓	✓	✓	✓	✓	✓	✓	✓	✓	N/A	✓	High
Nimptsch 2010 [108]	N/A	N/A	✓	✓	✓	✓	✓	✓	✓	N/A	✓	High
Nitter 2014 [50]	×	✓	✓	✓	✓	✓	✓	✓	✓	N/A	✓	High
Norat 2005 [28]	N/A	N/A	✓	✓	✓	✓	✓	✓	✓	N/A	✓	High
Pala 2009 [71]	N/A	N/A	✓	✓	✓	✓	✓	✓	✓	N/A	✓	High
Pala 2011 [32]	N/A	N/A	✓	✓	✓	✓	✓	✓	✓	N/A	✓	High
Papadimitriou 2020 [131]	?	✓	✓	✓	✓	✓	✓	✓	✓	N/A	✓	High
Park 2009 [43]	N/A	N/A	✓	✓	✓	✓	✓	✓	✓	N/A	✓	High
Perez-Cornago 2017 [112]	N/A	N/A	✓	✓	✓	✓	✓	✓	✓	N/A	✓	High
Perez-Cornago 2020 [120]	N/A	N/A	✓	✓	✓	✓	✓	✓	✓	N/A	✓	High
Price 2010 [121]	✓	✓	✓	✓	✓	✓	✓	✓	✓	N/A	✓	High
Rohrmann 2006 [105]	N/A	N/A	✓	✓	✓	✓	✓	✓	✓	N/A	✓	High
Rohrmann 2008 [116]	N/A	N/A	✓	✓	✓	✓	✓	✓	✓	N/A	✓	High
Romieu 2012 [85]	N/A	N/A	✓	✓	✓	✓	✓	✓	✓	N/A	✓	High
Romieu 2015 [77]	N/A	N/A	✓	✓	✓	✓	✓	✓	✓	N/A	✓	High
Romieu 2017 [91]	N/A	N/A	✓	✓	✓	✓	✓	✓	✓	N/A	✓	High
Rothwell 2020 [51]	×	✓	✓	✓	✓	✓	✓	✓	✓	N/A	✓	High
Sen 2019 [117]	N/A	N/A	✓	✓	✓	✓	✓	✓	✓	N/A	✓	High
Sieri 2008 [94]	N/A	N/A	✓	✓	✓	✓	✓	✓	✓	N/A	✓	High
Sieri 2013 [86]	N/A	N/A	✓	✓	✓	✓	✓	✓	✓	N/A	✓	High
Sieri 2015 [33]	N/A	N/A	✓	✓	✓	✓	✓	✓	✓	N/A	✓	High
Spencer 2009 [87]	N/A	N/A	✓	✓	✓	✓	✓	✓	✓	N/A	✓	High
Steinbrecher 2010 [128]	×	✓	✓	✓	✓	✓	✓	?	✓	N/A	✓	High
Stepien 2017 [52]	×	✓	✓	✓	✓	✓	✓	?	✓	N/A	✓	High
Suzuki 2009 [113]	N/A	N/A	✓	✓	✓	✓	✓	✓	✓	N/A	✓	High
Thiebaut 2001 [92]	N/A	N/A	✓	✓	✓	✓	✓	✓	✓	N/A	✓	High
Tjønneland 2007 [89]	N/A	N/A	✓	✓	✓	✓	✓	✓	✓	N/A	✓	High
Travis 2008 [75]	×	✓	✓	✓	✓	✓	✓	✓	✓	N/A	✓	High
Travis, Spencer 2009 [127]	?	✓	✓	✓	✓	✓	✓	✓	✓	N/A	✓	High
Travis, Crowe 2009 [126]	✓	✓	✓	✓	✓	✓	✓	✓	✓	N/A	✓	High
Trichopoulou 2010 [73]	N/A	N/A	✓	✓	✓	✓	✓	✓	✓	N/A	✓	High
van Duijnhoven 2009 [21]	N/A	N/A	✓	✓	✓	✓	✓	✓	✓	N/A	✓	High
van Gils 2005 [68]	N/A	N/A	✓	✓	✓	✓	✓	✓	✓	N/A	✓	High
Vece 2015 [61]	N/A	N/A	✓	✓	✓	✓	✓	✓	✓	N/A	✓	High
Ward 2010 [59]	✓	✓	✓	✓	✓	✓	✓	✓	✓	N/A	✓	High
Ward 2016 [29]	N/A	N/A	✓	✓	✓	✓	✓	✓	✓	N/A	✓	High
Ward 2019 [109]	N/A	N/A	✓	✓	✓	✓	✓	✓	✓	N/A	✓	High
Zamora-Ros 2013 [97]	N/A	N/A	✓	✓	✓	✓	✓	✓	✓	N/A	✓	High
Zamora-Ros 2017 [63]	N/A	N/A	✓	✓	✓	✓	✓	✓	✓	N/A	✓	High
Zamora-Ros 2018 [64]	N/A	N/A	✓	✓	✓	✓	✓	✓	✓	N/A	✓	High
Zuo 2018 [110]	×	N/A	✓	✓	✓	✓	✓	✓	✓	N/A	✓	High
**Criterion Score %**	44.7	100	100	100	100	100	100	93.6	100	N/A	100	

Note that the criterion score is calculated by dividing the number of studies meeting one criterion by the total number of studies. ✓: meet the methodological quality criterion; ×: not meet the methodological quality criterion.; ?: uncelar; N/A: not applicable.
